# A comprehensive approach to the molecular determinants of lifespan using a Boolean model of geroconversion

**DOI:** 10.1111/acel.12504

**Published:** 2016-09-09

**Authors:** Loic Verlingue, Aurélien Dugourd, Gautier Stoll, Emmanuel Barillot, Laurence Calzone, Arturo Londoño‐Vallejo

**Affiliations:** ^1^ Institut Curie CNRS, UMR3244 Telomere and Cancer Laboratory PSL Research University 75005 Paris France; ^2^ Department of Medical Oncology Institut Curie 75005 Paris France; ^3^ Institut Curie Mines Paris Tech, Inserm, U900 PSL Research University F‐75005 Paris France; ^4^ Sorbonne Paris Cité Université Paris Descartes 12 Rue de l'École de Médecine 75006 Paris France; ^5^ Equipe 11 labellisée Ligue contre le Cancer INSERM U 1138 Centre de Recherche des Cordeliers 15 rue de l'Ecole de Médecine 75006 Paris France; ^6^ Université Pierre et Marie Curie 4 Place Jussieu 75005 Paris France; ^7^ UPMC Univ Paris 06 CNRS, UMR3244 Sorbonne Universités 75005 Paris France

**Keywords:** Boolean modeling, lifespan expansion, *in silico* drug screening, rapamycin, type 2 diabetes mellitus, cancer

## Abstract

Altered molecular responses to insulin and growth factors (GF) are responsible for late‐life shortening diseases such as type‐2 diabetes mellitus (T2DM) and cancers. We have built a network of the signaling pathways that control S‐phase entry and a specific type of senescence called geroconversion. We have translated this network into a Boolean model to study possible cell phenotype outcomes under diverse molecular signaling conditions. In the context of insulin resistance, the model was able to reproduce the variations of the senescence level observed in tissues related to T2DM's main morbidity and mortality. Furthermore, by calibrating the pharmacodynamics of mTOR inhibitors, we have been able to reproduce the dose‐dependent effect of rapamycin on liver degeneration and lifespan expansion in wild‐type and HER2–neu mice. Using the model, we have finally performed an *in silico* prospective screen of the risk–benefit ratio of rapamycin dosage for healthy lifespan expansion strategies. We present here a comprehensive prognostic and predictive systems biology tool for human aging.

## Introduction

New translational tools are required to implement our increasing understanding of the molecular determinants of age‐related diseases into clinical interventions (Fontana *et al*., 2014). Aging is an organismal phenomenon associated with the progressive accumulation of senescent cells in tissues. This is a consequence of both an increase in pro‐senescence signals and an impairment in the senescent cells' clearance (López‐Otín *et al*., 2013). Cell senescence, which corresponds to a permanent growth arrest, is thought to negatively impact tissue function and thus to drive the manifestation of age‐related diseases (Muñoz‐Espín & Serrano, 2014). Persistent telomeric or genomic damages, oncogene activation, epigenomic perturbations, and/or tumor suppressor inactivation are among the main triggers of cell senescence (Campisi, 2013). However, an integrative approach to validate the molecular mechanisms that control S‐phase entry and transient/permanent cell growth arrest remains elusive (Piano & Titorenko, 2015). Furthermore, measuring the impact of the cell senescence on disease outcome represents a major clinical challenge.

The pharmaceutical inhibition of mTOR (comprising mTORC1 and/or mTORC2) is the most reproducible intervention to expand lifespan in preclinical models and correlates with a reduction of cell senescence proportions in many species including humans (Harrison *et al*., 2009; Anisimov *et al*., 2010; Popovich *et al*., 2014; Warner, 2015). Still, the pro‐proliferative activity of mTORC1 is conceptually difficult to reconcile with its role in permanent growth arrest. Moreover, a prophylactic mTOR inhibition may inevitably expose individuals to well‐known treatment's toxicities (MacDonald & RAPAMUNE Global Study Group, 2001; Mahé *et al*., 2005; Ferté *et al*., 2011). Modeling the dose‐related benefits and risks of rapamycin treatment in the molecular network that controls senescence could therefore contribute to healthy lifespan expansion strategies for humans.

Geroconversion is a specific type of cell senescence resulting from the inappropriate activation of growth signals in nonproliferative cells. At the molecular level, several studies have reported that the co‐activation of mTORC1 and CDKN1A (p21) is necessary to trigger geroconversion (Astle *et al*., 2012; Hasty *et al*., 2013; Blagosklonny, 2014). While AKT is both responsible for the activation of mTORC1 and the inhibition of CDKN1A's nuclear functions, questions persist about how mTORC1 and CDKN1A activations can occur concomitantly (Zhou *et al*., 2001). We have hypothesized that the numerous regulatory feedback loops in the PI3K/AKT/mTOR pathway may be responsible for the decoupling of mTORC1 and CDKN1A activities from AKT regulation thus allowing geroconversion to occur.

To test this hypothesis, we have first built a network of the molecular mechanisms involved in geroconversion and S‐phase entry. Then, by translating it into a Boolean model, we have been able to precisely reproduce the level of cell senescence in tissues from humans and mice with type‐2 diabetes mellitus (T2DM) compared to normal. Next, we have simulated the effect of rapamycin on lifespan expansion reported in the three largest longevity studies in mice for different genetic background and rapamycin doses. Prompted by the coherence of our results with experimental published data, we have finally proposed a prospective screen of the effect of rapamycin on lifespan expansion, balanced with the toxicity of rapamycin on oral mucosa. With this study, we aim at providing a comprehensive view of the molecular network of geroconversion to help defining precise therapeutic strategies.

## Results

### Construction of the mathematical model

**Figure 1:**
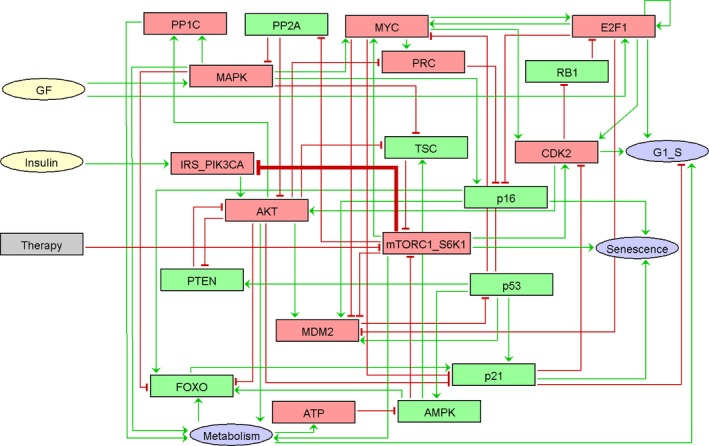
The influence network of geroconversion. The model has been built from the literature. Inputs are growth factors (GF) and insulin. Outputs are senescence, S‐phase entry (G1_S), and metabolism. Red nodes are pro‐proliferative factors, green nodes are antiproliferative factors, green arrows represent activations, and red arrows represent inhibitions. The thick edge concerning IRS_PIK3CA inhibition by mTORC1_S6K1 defines the type diabetes model. The normal model is identical in everything but without this thick edge. The gray node ‘Therapy' represents mTOR inhibitors. The exhaustive list of literature references for the molecular network of geroconversion is in Appendix S1.

After selecting the key genes participating in the pathways we wished to describe, we gathered known facts about the regulations of these genes from the literature (Table S1, Supporting information). The network that recapitulates this information is of the form of an influence network where nodes are genes, complexes, or processes, and edges are positive or negative influences. To build the molecular network that regulates geroconversion, we have started with the well‐documented regulations between the PI3K/AKT/mTOR and the p53/CDKN1A pathways (Hasty *et al*., 2013). We have added a simplified network of the cell cycling regulatory system to obtain a coherent behavior of the S‐phase entry readout (Aguda & Algar, 2003). We have then enriched the network with influences described in the literature about the constitutive regulations of the PI3K/AKT/mTOR pathway (Table S1). We have deliberately limited our model to these pathways to specifically analyze the impact of the metabolism signaling on cell cycling and senescence. By analyzing the resulting network, we have identified numerous feedback loops that represent constitutive regulations (Appendix S1, Supporting information). We have also identified a context‐specific circuit. Indeed, it has been described that excessive glucose uptake and obesity result in both insulin pathway‐dependent and pathway‐independent mTORC1 and S6K1 activity (Laplante & Sabatini, 2012). The persistent activity of S6K1 (mTORC1_S6K1 in the model) is known to negatively regulate the upstream insulin receptor substrate 1 (noted IRS1_PIK3CA in the model; Laplante & Sabatini, 2012). This inhibitory influence is suspected to be the cause of insulin resistance that defines type‐2 diabetes mellitus (T2DM; Laplante & Sabatini, 2012). We have therefore split the model into a *normal* model without this feedback and a *T2DM* model encompassing it (Fig. 1). The network was then translated into a Boolean model by adding logical rules to each of the variables of the two versions of the model and was simulated stochastically (see Materials and methods). One of the main advantages of Boolean models is that there are almost no parameters to tune. To simulate the model stochastically, we set all the initial conditions as random. The time‐dependent probabilities of the nodes' activities were obtained using MaBoSS (Stoll *et al*., 2012), by setting the initial probability of the nodes' activities to 0.5. Some of the variables represent ‘phenotypes': They integrate the activity of several nodes and they can be understood as readouts of the model. When the model is perturbed, these probabilities change and it is this particular change that is studied, compared to experimental data, and interpreted qualitatively.

### T2DM increases geroconversion

**Figure 2:**
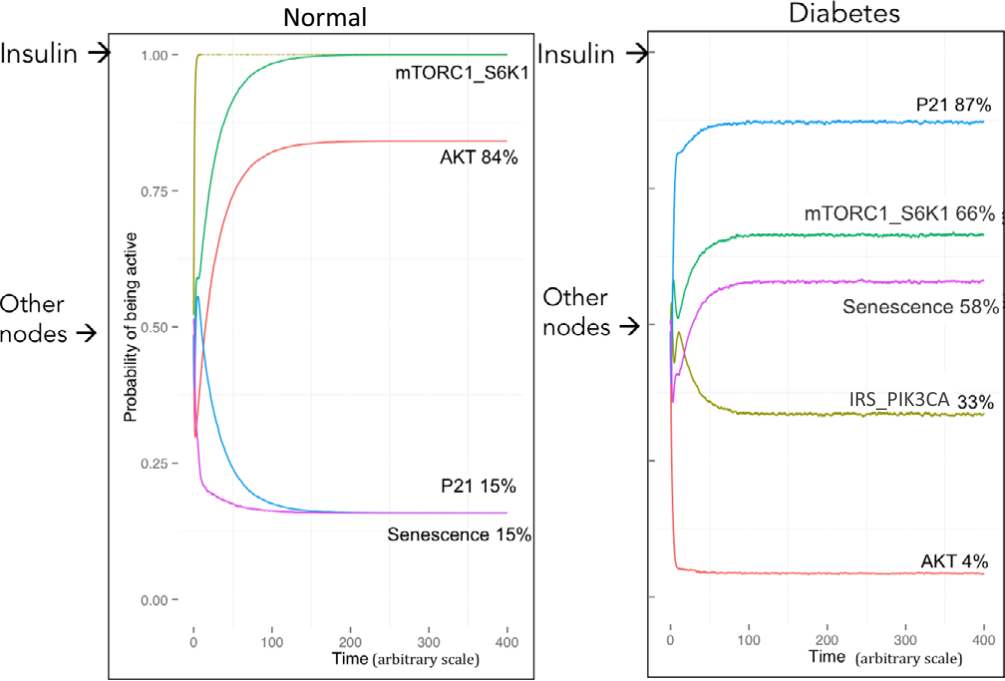
Simulations of the normal and the diabetes models. mTORC1_S6K1, AKT, senescence, and p21 are plotted using MaBoSS: The percentages represent the probability for a node of being active in time (arbitrary scale). Initial states of every variable simulated from the model are defined as random (‘Other nodes→‘) except for the insulin input defined=1 (‘Insulin→ ‘). (Left panel) Simulations for the normal model; (right panel) simulations for the diabetes model.

To assess the explanatory power of our model, we have initially evaluated how the influence responsible for the T2DM model could reproduce biological observations. As previously mentioned, the initial states of each variable of the model have been set with a probability of activity of 0.5 to fully cover all possible conditions. Concerning the inputs of the model (growth factors – GF, and insulin), we have observed that the predictions fit better the biology when the insulin node is permanently activated and the GF node is random. Importantly, this is consistent with the high blood concentrations of insulin during the day in humans (Daly *et al*., 1998). The main effect of the T2DM‐specific influence can be summarized as follows: In the normal model, IRS_PIK3CA is only regulated by insulin (IRS_PIK3CA active 100% of the time, same as insulin; Fig. 2, left panel). However, in the T2DM model, the IRS_PIK3CA activity depends on both insulin and mTORC1_S6K1 and thus is less active under the same insulin input, validating the driving effect of this functional feedback on insulin resistance (IRS_PIK3CA active 33% of the time for 100% constant insulin activity; Fig. 2, right panel). The consequence of a weaker activity of IRS_PIK3CA in response to high insulin level is a reduction of AKT activity, and as a consequence, a much stronger activation of CDKN1A. Meanwhile, mTORC1_S6K1 activity is partially conserved, creating a favorable situation for mTORC1_S6K1 and CDKN1A to trigger geroconversion. Indeed, there is a fourfold increase of the mean probabilities of senescence in the T2DM model compared to the normal model (Fig. 2).

**Figure 3:**
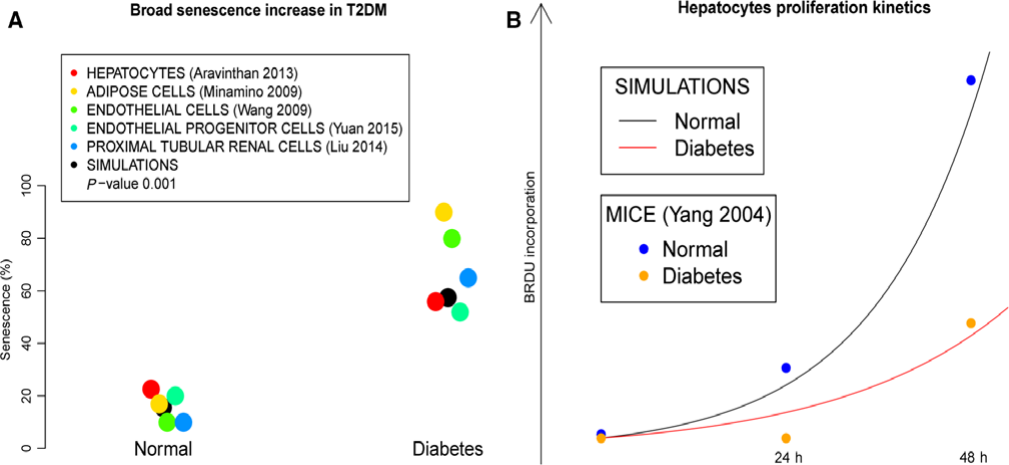
Comparison of the phenotypes between the normal and diabetes models. (A) The probabilities of senescence in the normal and diabetes models (black dots) reproduce the reported measurements of cell senescence proportions in a variety of tissues for the same conditions (color dots). (B) The proliferation kinetics deduced from the probabilities of S‐phase entry (G1_S) and senescence in the normal and diabetes models (black and red lines) reproduce the reported hepatocytes proliferation kinetics for the same conditions.

The activity of the senescence output obtained from the model was then compared to the proportions of cell senescence reported in the literature for normal and diabetic humans and mice. The method in study selection is described in the Appendix S2. Cell types such as hepatocytes, endothelial cells, renal cells and adipocytes are highly relevant for T2DM clinical complications in humans (Wang *et al*., 2009; Minamino *et al*., 2009; Aravinthan *et al*., 2013; Liu *et al*., 2014; Yuan *et al*., 2015). By simulating the model in the conditions reported below, we observed that the probabilities of senescence calculated from the simulations of the normal model perfectly coincide with the proportions of cell senescence reported for nondiabetic humans and mice (Fig. 3A). As for the diabetic model, the probabilities precisely reproduce the difference in the cell senescence proportions for diabetic humans and mice (Fig. 3A). With the model, we have computed the cell proliferation kinetics from the probabilities of S‐phase entry and corrected by the probabilities of senescence (see Materials and methods for equation of cell proliferation). For both the normal and diabetic models, the simulations also efficiently reproduce the proliferation kinetics of hepatocytes as evaluated by BrdU incorporation for normal and T2DM mice (Fig. 3B), further reinforcing the validity of our results (Yang *et al*., 2004).

The *in silico* simulations of this set of coherent biological observations suggest that the network structure we propose for geroconversion can be considered reliable. We have therefore frozen the model in its present configuration and have performed prospective simulations. We have searched the literature for comparable biological or clinical studies to find relevant biological interpretations for our results. (Appendix S2). For instance, our model predicts a 3.8 higher level of senescence in the T2DM model, which approximates the 3 times faster whole brain volume shrinkage detected in patients with diabetes. This result also approximates the 4.7‐fold increase in beta‐galactosidase‐positive (i.e. senescent) beta islet area found in mice with high‐fat diet, but with lower basal proportions (studies discussed in the Appendix S2). In all, increased geroconversion in patients with T2DM could be proposed as a unifying mechanism for T2DM‐related tissue complications.

Bona fide predictions of the normal and T2DM models also suggest an altered cell metabolism induced by T2DM. Cell metabolism is usually defined by the utilization of nutrients for energy production in the mitochondria, necessary for cell cycling and tissue function. In our model, the variable ‘Metabolism' corresponds to glycolysis, glycogenesis, and protein synthesis and indirectly controls ATP production, cell cycling, and FOXO activity (Table S1). Defective metabolism observed in T2DM effectively drives muscle loss (sarcopenia) and impacts functional capacities in the elderly (Leenders *et al*., 2013). Compared to the normal model, the simulation of the T2DM model induces a 21% reduction in metabolism (72% and 51% metabolism for the normal and T2DM models, respectively). This rate is strikingly close to the 21–28% skeletal muscle loss observed in T2DM mice, compared to normal (Wang *et al*., 2006). While a similar tendency has been observed in humans, raw data for metabolism change are not accessible for comparisons (Leenders *et al*., 2013; Larsen *et al*., 2015).

### How can T2DM induce both senescence and cancer?

**Figure 4:**
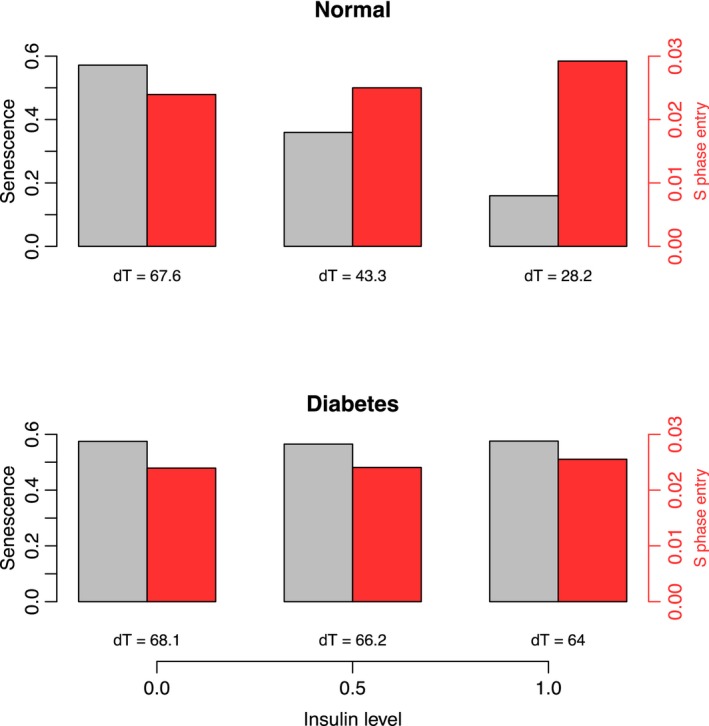
Proliferation kinetics deduced from the probabilities of S‐phase entry (G1_S) and senescence in the normal models under an increasing initial activity of the Insulin node. The probabilities of S‐phase entry (G1_S) are represented by a red bar and the probability of senescence by a gray bar. The ratios of the doubling times (dTs, see below) in the normal model reproduce the cancer‐related mortality increase for hyperinsulinemia (dT_insulin_1_/dT_insulin_0.5_ = 67.6/43.3 = 1.56; and dT_insulin_1_/dT_insulin_0_ = 67.6/28.2 = 2.39) The proliferation kinetics are deduced from the probabilities of S‐phase entry (G1_S) and senescence by the calculation of the dT with the formula: dT = log(2)/(G1_S ‐ G1_S*Senescence). The formula for the curves is f(x)  = exp(G1_S *x‐ G1_S*Senescence*x).

Patients with diabetes have a significant increase in cancer incidence and cancer‐related mortality (Tsilidis *et al*., 2015). Nevertheless, it may appear paradoxical that T2DM increases senescence, a well‐known tumor suppressive program for the cell, decreases cell metabolism, and concomitantly increases tumor cell proliferation. It has been postulated that peripheral insulin resistance induces a compensatory hypersecretion of insulin by the pancreas. Indeed, the largest epidemiological studies on this subject have identified hyperinsulinemia (or circulating C‐peptide or proinsulin) as a specific prognostic factor for cancer‐related mortality (Ma *et al*., 2008; Wolpin *et al*., 2009; Perseghin *et al*., 2012; Walraven *et al*., 2013). We have therefore simulated the normal and T2DM models as previously described, but with increasing levels of insulin (for each simulation, initial condition for insulin set to 0, 0.5 and 1) as the unique changing parameter. For each simulation, we have estimated the proliferation kinetics by calculating the doubling time (dT) from the probability of S‐phase entry (G1_S) corrected by the probability that the cells enter senescence (see Materials and methods). We have observed that in the normal model, increasing levels of insulin proportionally promote cell proliferation (Fig. 4, in red in the upper panels). The ratios of dT under hyperinsulinemia (insulin level = 1) compared to the 0.5 or 0 insulin levels are 1.56 and 2.39, respectively (dT_insulin_1_/dT_insulin_0.5_ = 67.6/43.3 = 1.56; and dT_insulin_1_/dT_insulin_0_ = 67.6/28.2 = 2.39). This is very close to the hazard ratios for cancer‐related mortality concerning patients with hyperinsulinemia, ranging between 1.62 and 2.38 (see Materials and methods for details; Walraven *et al*., 2013; Perseghin *et al*., 2012; Wolpin *et al*., 2009; Ma *et al*., 2008). Concerning the T2DM model, insulin level barely modifies the calculated dTs (Fig. 4, lower panels). These de novo predictions shed a new light on the complex relation between T2DM, loss of tissue function, and cancers (detailed in Appendix S3). Our Boolean model is in agreement with the observations of T2DM‐induced senescence and the prognostic value of hyperinsulinemia in cancer. It could thus be used as a predictive tool for pharmacological targeting.

### Reproduction of the pharmacodynamics and the liver effect of mTOR inhibitors

**Figure 5:**
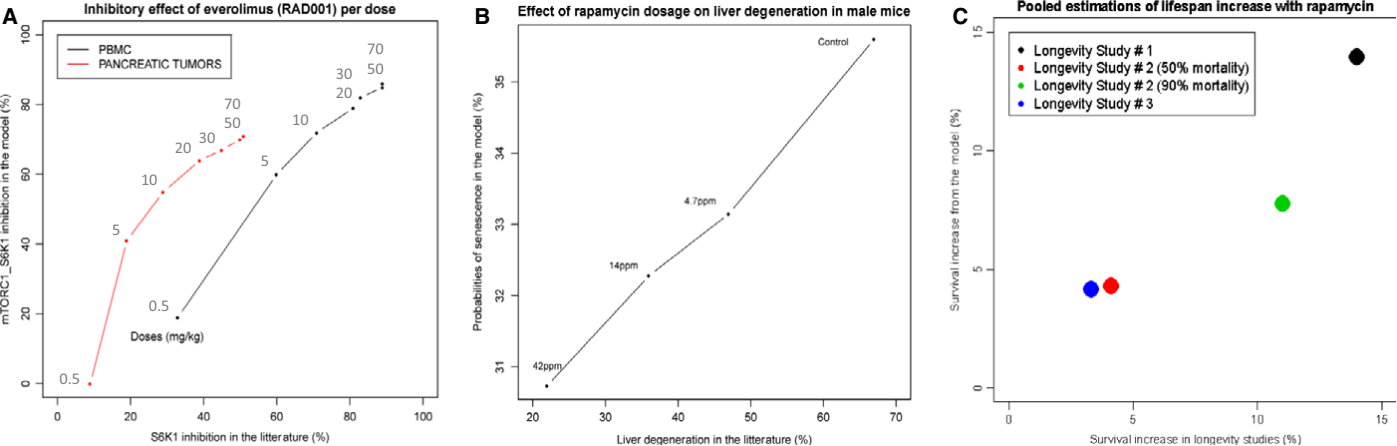
Simulations of the pharmacodynamics of mTOR inhibitors: predicted vs. published results. (A) For everolimus, simulated increasing levels of mTOR inhibition are proportional to the doses reported in (Boulay *et al*., 2004 ; Tanaka *et al*., 2008). The probabilities of mTORC1_S6K1 activities to the pharmacodynamics of everolimus in PBMC (peripheral blood mononuclear cells, Table S2; corresponding to the normal model) are compared to the pancreatic tumors (corresponding to the normal model with p53 loss of activity and MAPK gain of activity). Pearson correlation = 0.987, *P* value = 3.36e‐05 for PBMC and Pearson correlation = 0.926, *P* value = 0.00271 for tumors. (B) For rapamycin, simulated increasing levels of mTOR inhibition are proportional to the doses reported in (Wilkinson *et al*., 2012). Probabilities of senescence from the normal model are plotted with respect to the liver degeneration (corresponding to NAFLD) in male mice (Table S3): Pearson correlation = 0.997, *P* value = 0.00271. (C) Reproduction of the lifespan increase under rapamycin from 3 longevity studies. Longevity study #1: Simulation of intravenous administration of rapamycin at 2.24 mg kg^−1^day^−1^ for genetically heterogeneous female mice. Longevity study #2: Simulation of subcutaneous administration of rapamycin at 1.5 mg kg^−1^ 3 times a week for 2 weeks followed by a 2‐week break for HER2–neu mice. Longevity study #3: Simulation of subcutaneous administration of rapamycin at 0.45 mg kg^−1^, 3 times a week for 2 weeks followed by a 2‐week break for HER2–neu mice (Table S4 and Figs 3 and 4).

To account for a therapeutic intervention, we have introduced in the model a new variable that inhibits mTORC1_S6K1 (variable ‘Therapy' in Fig. 1), without any other change in the model or parameters. A single administration of an mTOR inhibitor has been simulated in the model by an initial peak of activity followed by a smooth decrease of the variable ‘Therapy', consistent with classical pharmacokinetics in mammals. The simulated slope for a single 5 mg administration of everolimus has been manually calibrated to fit the level of S6K1 inhibition at 168 hours measured in the peripheral blood mononuclear cells (PBMC; Boulay *et al*., 2004; Tanaka *et al*., 2008). By modifying the slope of decrease (rate down) of the variable ‘Therapy', it was possible to change its inhibition of mTORC1_S6K1 (the syntax and the mathematical formulae used to tune the parameters of the variable ‘Therapy' can be found in Materials and methods). Therefore, the effect of the 6 other treatment doses and control in PBMC of humans and/or mice have been prospectively simulated by modifying the slope of the variable ‘Therapy' proportionally to the dose modification (Table S2 and Fig. S1, Supporting information). The resulting probabilities of the inhibition of mTORC1_S6K1 activity replicate the effect of everolimus on the inhibition of S6K1 activity in humans and mice, validating this methodology to reproduce different doses of treatment (Pearson correlation = 0.987, *P* value = 3.36e‐05 for PBMC; Fig. 5A). It is also possible to simulate a gain or loss of function in the model. For pancreatic tumors, we have prospectively simulated the model with a p53 loss of function and MAPK gain of function (the most frequent alterations of pancreatic tumors) under the same doses of mTOR inhibitors. Again, we have observed an accurate reproduction of the pharmacodynamics of everolimus (Pearson correlation = 0.926, *P* value = 0.00271 for tumors). Interestingly, the saturating effect of high doses of everolimus reported by the authors has also been observed in our simulations: Above 30 mg, the delta of mTORC1_S6K1 inhibition is blunted (Fig. 5A).

Periodic administrations of the same dose of the drug have been simulated by a plateau of activity of the variable ‘Therapy' estimated from the residual activity at 24 h, which is consistent with classical pharmacokinetics in mammals. At the tissue level, Wilkinson and colleagues have analyzed the effect of a daily administration of rapamycin on age‐related liver degeneration, which corresponds to NAFLD (Wilkinson *et al*., 2012). Because the pharmacokinetics of everolimus and rapamycin are similar, the variable ‘Therapy' calibrated on a single 5 mg administration of everolimus has been used to calculate the pharmacodynamics of oral rapamycin at different doses (Materials and methods). We have deduced the probabilities of senescence from the three doses of rapamycin and the control (Table S3 and Fig. S2). Remarkably, the senescence probabilities highly correlate with the reported proportion of liver degeneration (Pearson correlation = 0.997, *P* value = 0.00271; Fig. 5B). Together with the previous observations, these data support that geroconversion of the cell is causal in NAFLD and can be efficiently prevented by rapamycin treatment in a dose‐dependent manner.

### Predicting lifespan expansion: the in silico contribution to rapamycin dosage

The effect of rapamycin on lifespan expansion is the most reproducible intervention for many species including mice, the latter being assessed in large ‘longevity studies'. Reasons for the selection of these studies are provided in the Appendix S3. The genetic background of the mice, rapamycin dose, schedules, and administration route of the three largest longevity studies fitted for comparison had been precisely reproduced in our simulations (Materials and methods; Harrison *et al*., 2009; Anisimov *et al*., 2010; Popovich *et al*., 2014). We have hypothesized that the probabilities of senescence from the model could approximate the probability of mortality in mice cohorts. Therefore, the probabilities of senescence for each simulation have been plotted by a negative, linear function of time, from 100% to 0% (Figs S3 and S4; Table S4). The differences between the senescence proportions in the ‘treatment' and ‘no treatment' groups at 90%, and for one study at 50%, mortality, have been compared to the corresponding data reported in the three longevity studies. The *in silico* quantifications of lifespan increase with rapamycin coincide with the ratios of mice survival (Fig. 5C). This result supports the high predictive value of the therapeutic simulations of the Boolean model and warrants its use to perform an *in silico* screen of the effect of rapamycin on lifespan expansion.

As the model efficiently predicts the senescence probabilities in humans and mice (Fig. 3A), we aimed at specifying the modalities for human prophylactic rapamycin treatment. From this perspective, it appears important to concomitantly consider the drug's toxicity. Oral ulcer is the best‐studied and most frequent dose‐related adverse event of mTOR inhibitors. It is one of the earliest ones, and perhaps the one that degrades the most the quality of life of treated patients. We have therefore pooled together the data about the dose‐dependent oral ulcers incidence reported in three human studies for everolimus and rapamycin (MacDonald AS & RAPAMUNE Global Study Group, 2001; Mahé *et al*., 2005; Ferté *et al*., 2011). We have found a linear relation between the published doses of mTOR inhibitors (in log scale) and oral ulcers appearance (Fig. S5). From this relation, 100% oral ulcers occur for 0.21 mg kg^−1^ day^−1^ of everolimus or rapamycin (Fig. S6). Interestingly, we have observed that the estimated doubling time from our model fits the oral ulcer appearance from the literature in a dose‐related manner (Pearson correlation = 0.942; Fig. S6A and B). Furthermore, the physiological doubling time of gastrointestinal mucosa and hematopoietic cells, 48 h, is fitted by our model (Odorico *et al*., 2001; Fowler *et al*., 2003; Yang *et al*., 2011, p.4). These observations underlie the great ranges of possibilities the model offers for therapeutic management of mTOR inhibitors.

**Figure 6:**
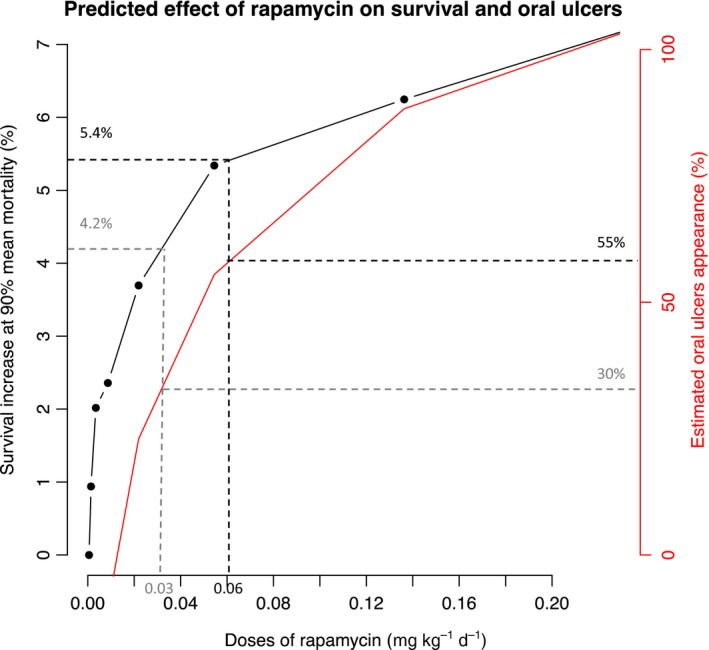
Predicted effect of rapamycin on survival and oral ulcer from the model. Simulated screen of rapamycin doses up to 0.20 mg kg^−1 ^day^−1^ to quantify the survival increase at 90% mean mortality. The dose‐related oral ulcer appearance has been deduced from published data (Figs S5 and S6). Two doses are represented with their risk–benefit ratio (dashed gray and black line).

We have therefore performed a screen of the doses of rapamycin between 0 and 0.21 mg kg^−1 ^day^−1^ to predict the lifespan increase from the model. The lifespan increase with rapamycin has been deduced from the difference with the control group at mean 90% mortality. The estimations from the model have identified a 6.8% lifespan expansion for a dose corresponding to 100% incidence of oral ulcers of all grades (Fig. 6). Interestingly, the risk–benefit ratio varies with the dose. For 0.06 mg kg^−1 ^day^−1^, we predict with the model a 5.4% survival increase with 55% oral ulcers appearance, which corresponds to a gain of 4 years in a population with 80‐year‐old lifespan expectancy. A dose of 0.03 mg kg^−1^ day^−1^ rapamycin still provides an interesting 4.2% predicted lifespan expansion while provoking less oral ulcer appearance (30%). These results could help refining the dose of mTOR inhibitors for future clinical trials.

It is still debated whether rapamycin expands lifespan *per se* or if it reduces age‐related diseases such as T2DM or cancers. In HER2–neu mice, Anisimov and colleagues have shown a reduction in cancer incidence for mice treated with rapamycin (Anisimov *et al*., 2010). With the model, the percentage reduction in cell proliferation between the control and the rapamycin model with MAPK hyperactivation reproduces this difference: 33.7% reduction of tumor‐bearing mice in the literature vs. 32% in the model (Fig. S7). This result brings insight in the coexisting roles of mTORC1 inhibition in both lifespan expansion *per se* and cancer prevention.

## Discussion

According to the World Health Organization, type‐2 diabetes mellitus currently concerns 347 million people worldwide, which is projected to increase to more than 550 million and to represent the 7th leading cause of death by 2030 (http://www.cdc.gov/diabetes/home/index.htlm;http://www.who.int/en/). This highlights the need for precocious prevention strategies. With a generic model of geroconversion, we have been able to precisely reproduce the increase in cell senescence related to the greatest morbidity and mortality for patients with T2DM: cardiovascular complications, nephropathy, and NAFLD (Fig. S8). Consistently, our model correlates to the reported faster whole brain volume shrinkage and metabolism defects in patients with T2DM, while providing molecular scenarios to explain the higher incidence of tissue‐specific cancers in that population. Our model also efficiently reproduces the hazard ratios reported for cancer‐related mortality in patients with hyperinsulinemia. The correct *in silico* results of such diverse physiopathological behaviors reported across multiple large biological and clinical studies allow us to conclude that the molecular network we have built does reflect one of the essential biological processes of the cell cycle control in mammals, and should be used as a basis to explore further hypotheses.

The importance of cell senescence triggered by mTORC1 activity has been recently highlighted from the rapamycin studies. The studies we used for the parameterization of the model about T2DM induced senescence focused on cases where the effect of the classical causes of senescence was minimized, namely persistent telomeric or genomic damages, oncogene activation, epigenomic perturbations, and/or tumor suppressor inactivation. Several topic‐related studies were excluded from our work for infeasible comparison (Appendix S2 and S3). We identified two studies that have reported a lower senescence proportions in the normal and T2DM conditions than in the other transorgan studies, however conserving a similar difference induced by T2DM compared to our model. Unfortunately, the authors do not address this inadequacy in their publications; therefore, we have not used them in Fig. 3A. Concerning the epidemiological studies on cancer prognosis and the longevity studies on mice, we aimed at being exhaustive, as long as the studies were comparable to our simulations with a reliable statistical power (Appendix S3). More comparisons and experimental reproductions would have reinforced the predictive power of our model. However, we decided to concentrate on a selected number of studies that had very large‐scale and high‐quality reported data.

Mathematical modeling of molecular networks is still an emerging field that aims at verifying and testing biological and pharmacological hypotheses. We provide in this study a new methodology to simulate the pharmacodynamics of everolimus and rapamycin. Using this methodology, we have calibrated the activity of the variable ‘Therapy' on a single 5 mg administration of everolimus and have deduced all the other dosages. We have define that cell senescence modulation under treatment could constitute a reliable surrogate marker of tissue function and lifespan. It has allowed us to reproduce the effect of these drugs on mice liver degeneration and lifespan with an unprecedented accuracy compared, to our knowledge, to the largest and most reliable longevity studies to date.

In the field of mathematical modeling, Boolean models rely on coarse‐grain descriptions rather than precise details of biochemical reactions involved in the processes we describe. This formalism allows us to minimize the number of parameters that need to be tuned, but this is carried out at the expense of the type of predictions that can be made. We coped with this issue by simulating the model with a modeling tool, MaBoSS, which performs stochastic simulations of the Boolean model and provides quantitative outputs by modulating a very limited amount of parameters (only one parameter was tuned here). Other types of formalisms, for example, chemical kinetics, could permit a more precise, or more qualitative analysis, but the very little amount of information on the reaction parameters compromises the realization of such mathematical models.

The fact that such a relatively simple molecular network is able to provide such a comprehensive and accurate view of very diverse biological and clinical observations may be surprising. At the same time, these observations support the view that our model is equilibrated and covers a fundamental mechanism for cell cycle control in mammals. Clearly, robust models must rely on strong experimental evidences used to construct the network. It seems important to permanently verify the coherence of the network during the construction phase, and to stick to well characterized mechanisms. Given the complexity of the biology of molecular interactions, the model has to maintain an equilibrium and therefore could suffer from too many details in particular modules thus impacting the whole network. Larger models shall express exponential complexity to keep on fitting the biology. Nevertheless, this first model of geroconversion together with our new methodology to simulate therapies may contribute to the understanding and management of major health problems related to aging, namely T2DM and cancers.

## Materials and methods

### Experimental design

Molecular interactions are necessary for signal transduction in the cell. We have manually curated the molecular signals in response to growth factors and insulin activation, from the literature (Table S1). The network obtained has been translated into a mathematical model using a logical formalism to calculate the probabilities of S‐phase entry and senescence. Thereby, the molecular conditions of published studies have been reproduced in the model to compare the *in silico* predictions to the biological phenotypes.

### Boolean modeling

Boolean logic, a discrete dynamical formalism, is based on a low‐resolution description of a molecular network. Nodes represent entities such as genes, proteins, complexes, and processes, and edges represent their mutual influences. Each node can be found in 2 states: 1 (on) or 0 (off). The activating or repressing influences on a target node are defined by the logical gates: AND, OR, NOT. Nodes can also be transiently or permanently forced into a Boolean state, which is used to simulate the effect of extracellular signals, or genomic alterations, for example. Various simulation strategies can be applied for Boolean models. First, we have computed the attractors of the Boolean model to define the stable states or limit cycles using GINsim (Naldi *et al*., 2009). Then, we have simulated the model stochastically to assess the probabilities of the phenotypes ‘senescence' or ‘G1_S' and of some internal variables of the model. These calculations were performed using MaBoSS software, which allows the stochastic simulations of Boolean models (Stoll *et al*., 2012). It computes the probabilities of activation of each node over continuous time by simulating a Markov process using the Gillespie algorithm. The model is freely available at the BioModels database (https://www.ebi.ac.uk/biomodels-main/) and GINsim (http://ginsim.org) repositories. MaBoSS script is also provided in this article (Appendix S4).

### T2DM data

Biological data that evaluate the impact of T2DM on tissues were retrieved from the literature with a particular attention on the most frequent T2DM tissue‐specific complications in the clinic (for the selection's rational see Appendix S2). Selected studies that have measured the senescence proportions in humans and mice have focused on hepatocytes from 70 patients, progenitor endothelial cells from 83 patients, differentiated endothelial cells from mice, proximal tubular renal cells from 30 patients, and adipose cells from mice, corresponding to the following T2DM clinical complications: NAFLD, cardiovascular complications, diabetic nephropathy, global inflammation, and aggravation of insulin resistance, respectively (Wang *et al*., 2006, 2009; Minamino *et al*., 2009; Aravinthan *et al*., 2013; Liu *et al*., 2014; Yuan *et al*., 2015). One of the studies also evaluates the BrdU incorporation in time of cells from obese and nonobese mice obtained by hepatic biopsy (Yang *et al*., 2004).

### Hyperinsulinemia and cancers' mortality

Four large epidemiological studies have evaluated the relation between hyperinsulinemia (or surrogate markers like circulating C‐peptide or proinsulin) and cancer‐related mortality. For unselected cancer types, high levels of circulating proinsulin or insulin are associated with hazard ratios of 2.01 and 1.62 for mortality in 438 and 2011 individuals, respectively (Perseghin *et al*., 2012; Walraven *et al*., 2013). For colorectal and prostate cancers, high levels of circulating C‐peptide are associated with hazard ratios of 1.87 and 2.38 for mortality in 373 and 2546 patients, respectively (Ma *et al*., 2008; Wolpin *et al*., 2009).

### Simulations protocol

To be compared with the biological data, the simulations have been performed assuming the following rules:

1. Every stochastic simulation has been pursued until a stable state of probabilities is reached, except when a specific time is necessary, for example for the therapy calibration.
2. Every node in the model has been simulated with a probability of initial activity of 0.5, except for the simulations of hyperinsulinemia (Insulin initial state = 1) and mTORC1 inhibition (Therapy initial state = 1).
3. The parameters ruling the interactions between the nodes (rate up and rate down) have been equally set = 1, and no rates have been fitted except for, and limited to 1) the variable therapy (rate used to simulate doses, described thereafter) and 2) the simulation of the p53 loss of function and the MAPK gain of function (MaBoSS script available in Appendix S4).
4. The curves of BrdU incorporation and cell proliferation kinetics have been arbitrary calculated by the exponential function of the probability of S‐phase entry (G1_S) corrected by the probability that the cells undergo senescence:
5. f(t)  = exp(G1_S*t ‐ G1_S*Senescence*t)
6. The estimations of the doubling time (dT) from the simulations have been arbitrary calculated with the formula: dT= log(2)/(G1_S ‐ G1_S*Senescence)

The experimental procedures of the three longevity studies have been reproduced in the simulations for the estimations of lifespan and carcinogenesis (Harrison *et al*., 2009; Anisimov *et al*., 2010; Popovich *et al*., 2014). The estimation of lifespan has been performed using the probabilities of senescence as a linear decreasing function in time, by: f(t)  = 1‐Senescence*t.

### Therapeutic simulations protocol

An input node ‘Therapy' that inhibits mTORC1_S6K1 has been added to the initial model and used to simulate the effect of everolimus and/or rapamycin (Fig. 1). The corresponding syntax in MaBoSS is as follows:





Node Therapy {

rate_up = $u_ Therapy;

rate_down = $d_ Therapy;

}

Node mTORC1_S6K1 {

logic = (!AMPK & !TSC);

rate_up = (@logic AND NOT Therapy) ? $u_mTORC1_S6K1: 0;

rate_down = @logic ? 0: $d_mTORC1_S6K1;

}





where $u_ Therapy and $d_ Therapy are assigned to a numeric value and correspond to the rate of increase ($u) or decrease ($d): These parameters are tuned to match the experimental observations.

We first set the initial state of Therapy to 1 to simulate the initial peak of treatment concentration. To calibrate the value of $u_ Therapy and $d_ Therapy, we used data from (Boulay *et al*., 2004; Tanaka *et al*., 2008). Authors have performed a single administration of several doses of everolimus to humans and rats and have measured their effects on S6K1 during 168 hours in PBMC (considered to be wild‐type for the simulations) and pancreatic tumors (considered to harbor a p53 loss of function and a MAPK gain of function for the simulations). The pharmacokinetics and pharmacodynamics reported for a single administration of 0.5 mg/kg everolimus have been considered as the reference dosage. Therefore, the parameter for the slope of decrease of the variable ‘Therapy' was tuned to obtain 19% of S6K1 inhibition at 168 hours ($u_ Therapy = 0.035 and $d_ Therapy = 1‐$u_ Therapy). The transformation factors from 0.5 mg kg^−1^ have been used to calculate the slope of the simulated therapy for other doses (Table S2).

As the blood pharmacokinetics of rapamycin is similar to the blood pharmacokinetics of everolimus for the same dose, the previous reference dosage was used to simulate several doses of rapamycin (http://www.ema.europa.eu/docs/en_GB/document_library/EPAR_-_Scientific_Discussion_-_Variation/human/000273/WC500046440.pdf). Senescence probabilities from the simulations of daily administration of rapamycin at 3 different doses were compared to the proportion of age‐related liver degeneration in mice (Wilkinson *et al*., 2012).

Furthermore, the effect of rapamycin on lifespan expansion in mice reported in 3 ‘longevity studies' was used for comparison. First, Harrison and colleagues have evaluated the oral administration of rapamycin at 2.24 mg kg^−1^ day^−1^ in 1916 genetically heterogeneous mice and matched controls and have found a 14% increase in survival at 90% mean mortality for female mice (Harrison *et al*., 2009). Second, Anisimov and colleagues have assessed the effect on lifespan and carcinogenesis of subcutaneous administration of rapamycin at 1.5 mg kg^−1^, 3 times a week for a period of 2 weeks followed by 2‐week intervals without rapamycin, on HER2–neu female mice. They have found 4.1% and 11% lifespan increase at 50% and 90% mean mortality, respectively (Anisimov *et al*., 2010). Third, Popovich and colleagues have used the same transgenic HER2–neu mice model to assess the effect on lifespan and carcinogenesis of rapamycin subcutaneous at lower doses, namely 0.45 mg kg^−1^, 3 times a week for 2 weeks followed by a 2‐week break (Popovich *et al*., 2014). They have found a mean 3.3% increase in lifespan from their 3 treatment groups. The subcutaneous administration has been simulated by a correction of 0.1 from the oral doses consistent with the known 10% bioavailability of subcutaneous rapamycin (Crowe *et al*., 1999).

### Statistics

The data analyses, statistic tests, and plots have been performed using the R software version 3.1.2 (2014‐10‐31). The correlations have been calculated with the Pearson test. Linear regressions and corresponding p‐values have been calculated with the function linear model (lm) of the package ‘stats'.

## Author contributions

LV designed research, performed research, contributed analytic tools, analyzed data, and wrote the manuscript. AD, GS, and EB contributed analytic tools. LC designed research, contributed analytic tools, and wrote the manuscript. AL designed research and wrote the manuscript.

## Funding

LV has received a one‐year grant from Institut Curie to perform this study. The Telomere and Cancer laboratory is ‘labellisé' Ligue. Work in this laboratory is also supported by ANR and INCa.

## Conflict of interests

None.

## Data and materials availability

https://www.ebi.ac.uk/biomodels-main/; Appendix S4.

## Supporting information


**Fig. S1** Simulations of single doses of everolimus on PBMC and pancreatic tumors.

**Fig. S2** Simulations of single and daily administrations of rapamycin on the dose‐related liver degeneration.

**Fig. S3** Simulations of single and daily administrations of rapamycin on the dose‐related lifespan expansion in mice.

**Fig. S4** Simulations of the survival increase with rapamycin.

**Fig. S5** Relation between rapamycin dose and toxicity.

**Fig. S6** mTOR inhibitors toxicity can be deduced from the estimated doubling times.

**Fig. S7** The anti‐proliferative effect of rapamycin.

**Fig. S8** Comprehensive overview of the tissue specific impact of type 2 diabetes (T2DM) in the spectrum of the predictions from our model.

**Table S1** List of literature references for the molecular network of geroconversion.
**Table S2** Details for the inhibitory effect of everolimus per dose in PBMC and pancreatic tumors.
**Table S3** Details for the effect of rapamycin dosage on liver degeneration in male mice.
**Table S4** Details for the pooled estimations of lifespan increase with rapamycin.
**Appendix S1** Constitutive regulations that can be found in the molecular network.
**Appendix S2** Rational for senescence studies selection.
**Appendix S3** Rational for cancer types & longevity studies selection.
**Appendix S4** MaBoSS script for the normal model presented in Fig 1 and containing the logical rules (without the T2DM feedback).
