# Supplementary figures and images for: A comprehensive approach to the molecular determinants of lifespan using a Boolean model of geroconversion
Source: Aging Cell. 2016 Sep 9;15(6):1018–26. doi: 10.1111/acel.12504 (PMC6398530; doi:10.1111/acel.12504)

PBMC

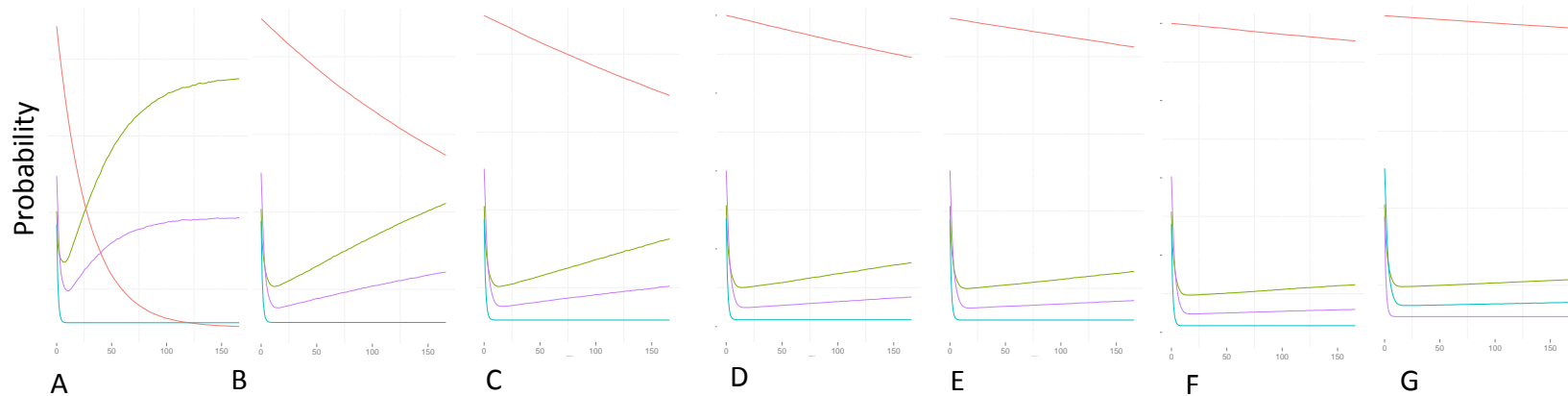

Pancreatic tumors

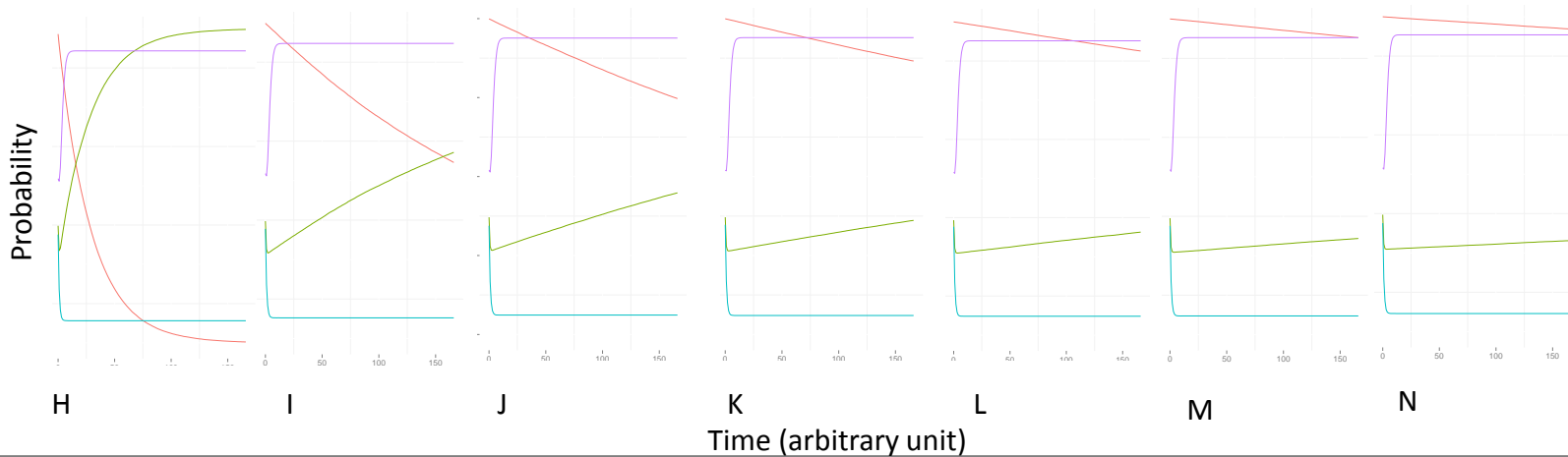

Therapy  
mTORC1\_S6K1  
Senescence  
G1\_S

Supplement: Supplementary file 1 — Fig. S1 Simulations of single doses of everolimus on PBMC and pancreatic tumors. [file ACEL-15-1018-s001.pdf]

Single dose

Probability

A

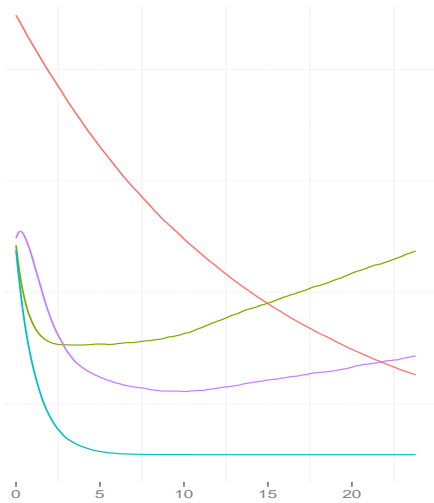

B

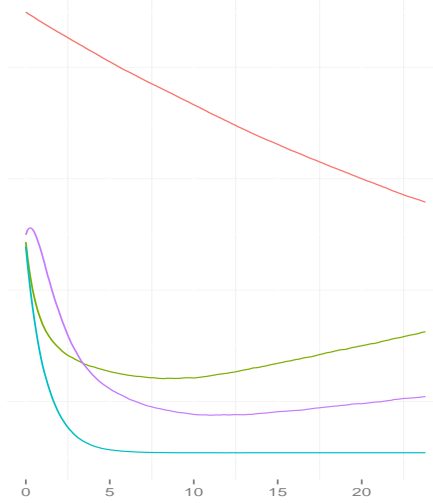

C

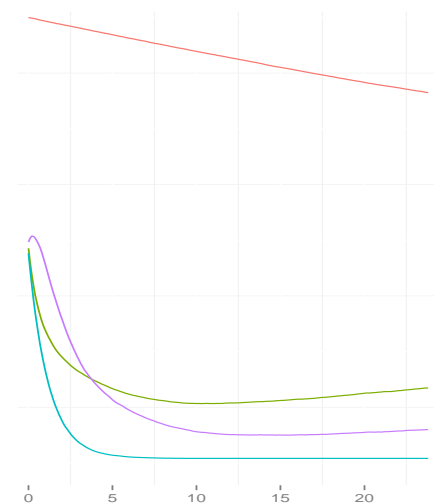

Daily dosage

Probability

D

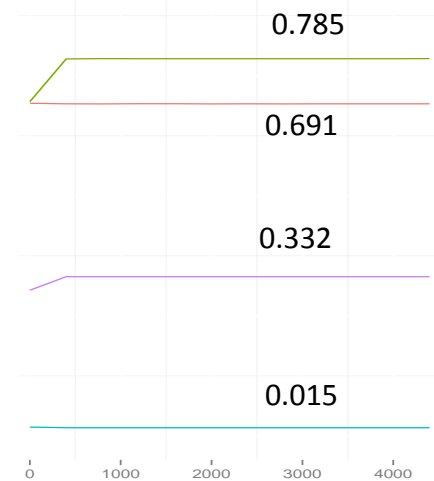

E

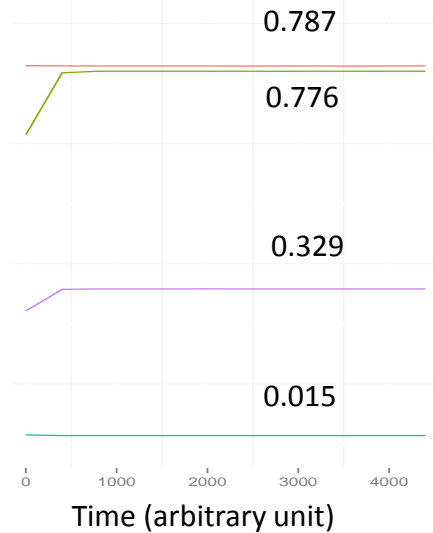

F

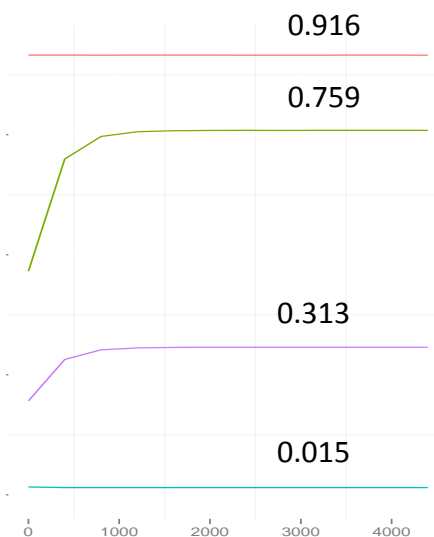

Therapy  
mTORC1\_S6K1  
Senescence  
G1\_S

Supplement: Supplementary file 2 — Fig. S2 Simulations of single and daily administrations of rapamycin on the dose‐related liver degeneration. [file ACEL-15-1018-s002.pdf]

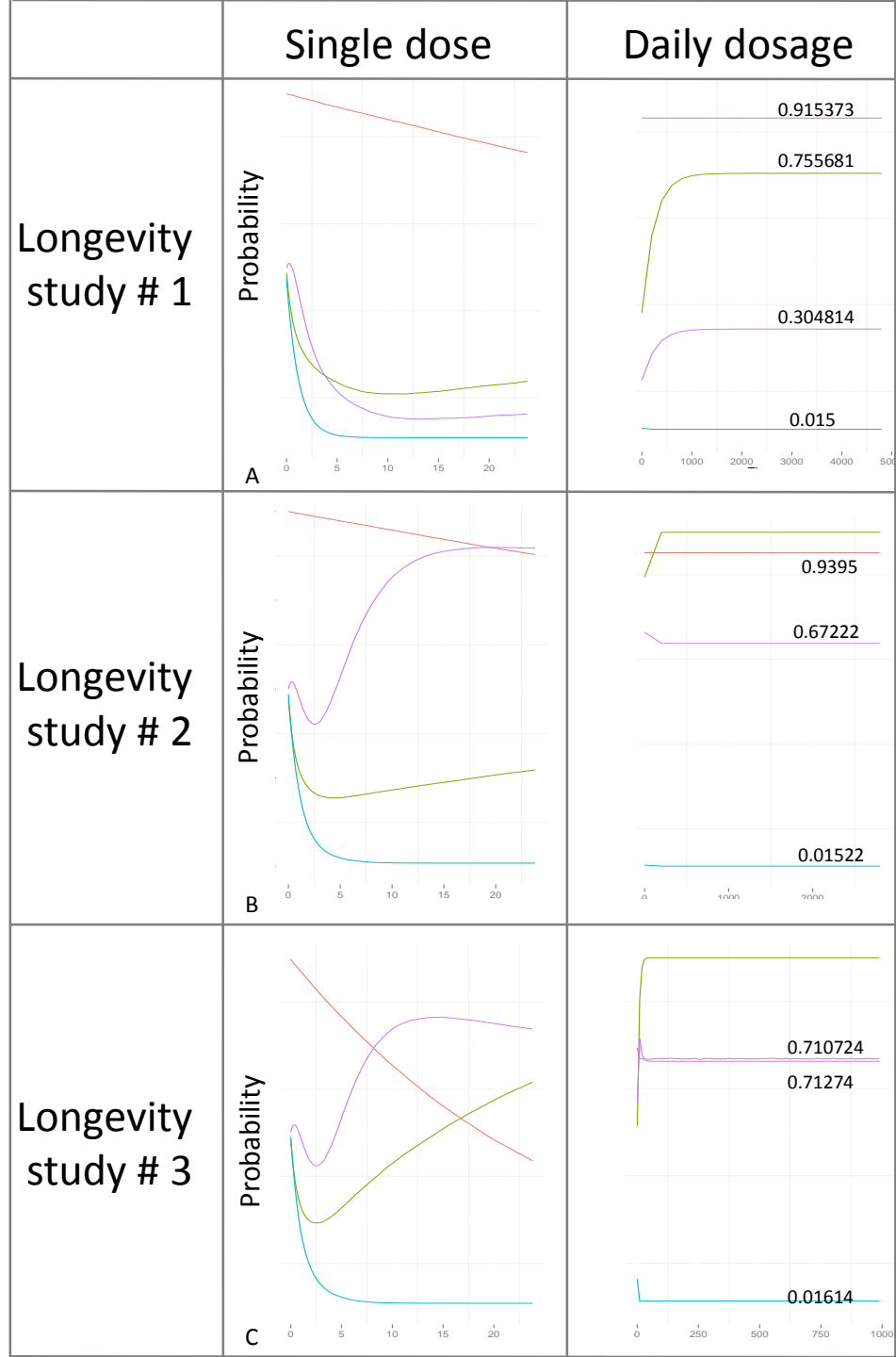

— Therapy  
— mTORC1\_S6K1  
— G1\_S  
— Senescence

Supplement: Supplementary file 3 — Fig. S3 Simulations of single and daily administrations of rapamycin on the dose‐related lifespan expansion in mice. [file ACEL-15-1018-s003.pdf]

### Rapamycin for WT mice

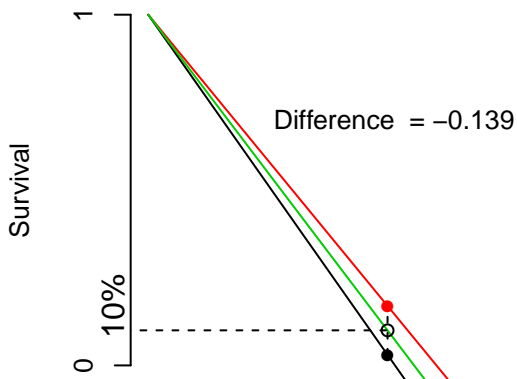

### High dose rapamycin: HER2-neu mice

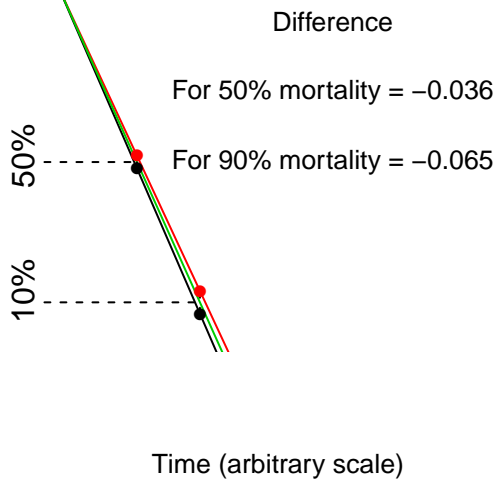

### Low dose rapamycin: HER2-neu mice

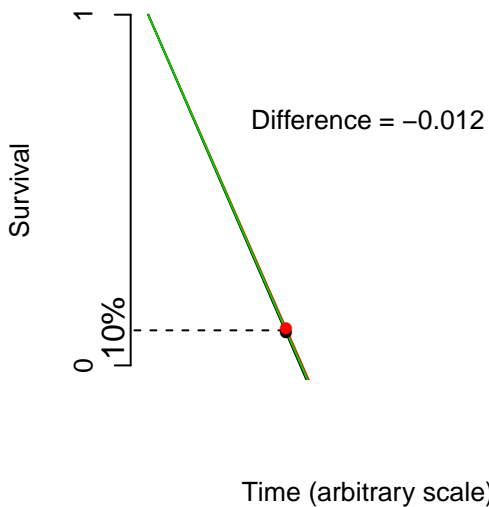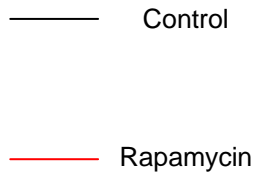

Supplement: Supplementary file 4 — Fig. S4 Simulations of the survival increase with rapamycin. [file ACEL-15-1018-s004.pdf]

## Relation between dose and toxicity

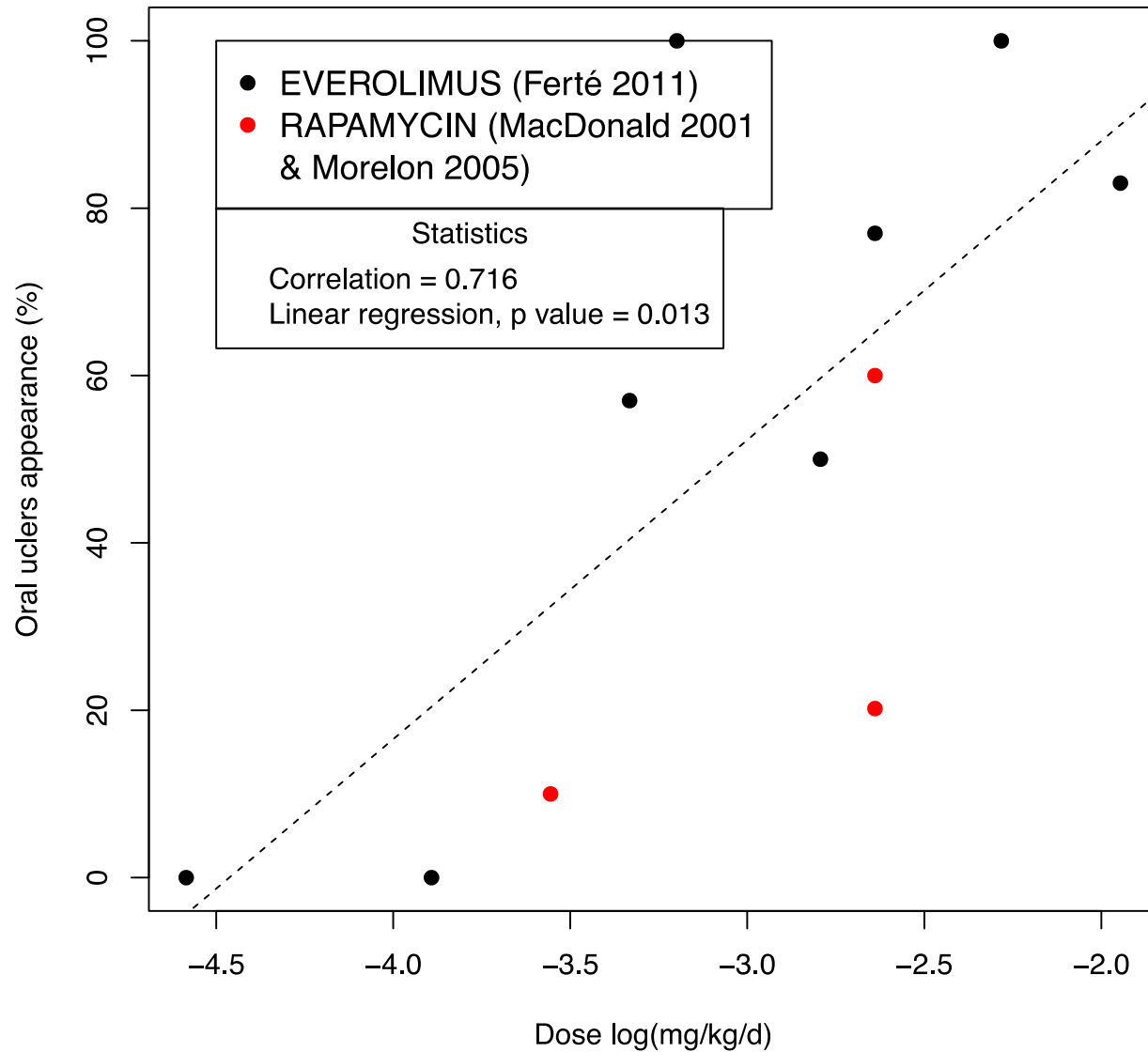

Supplement: Supplementary file 5 — Fig. S5 Relation between rapamycin dose and toxicity. [file ACEL-15-1018-s005.pdf]

## Effect of rapamycin on cell proliferation in HER2-neu mice

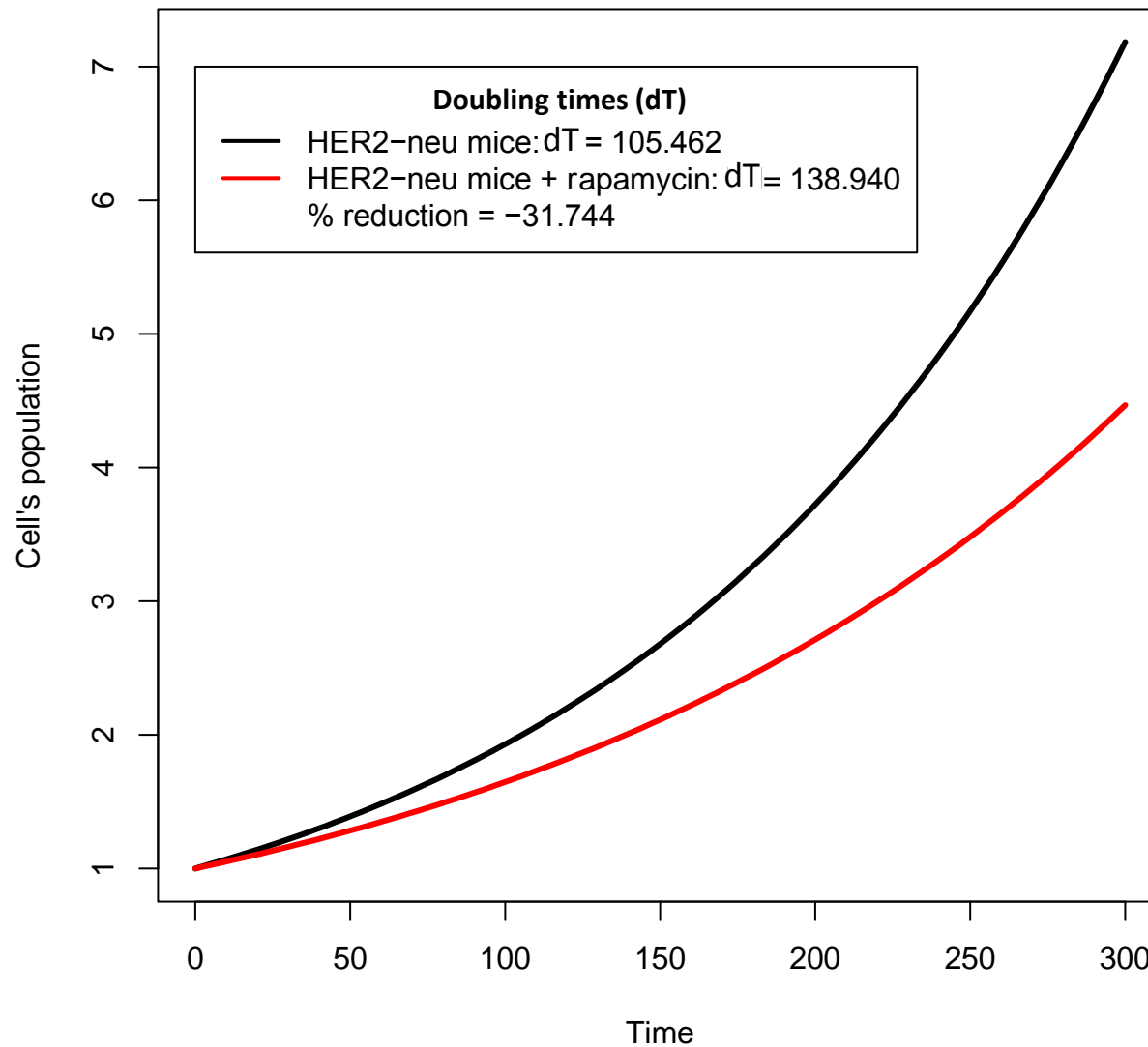

Supplement: Supplementary file 7 — Fig. S7 The anti‐proliferative effect of rapamycin. [file ACEL-15-1018-s007.pdf]

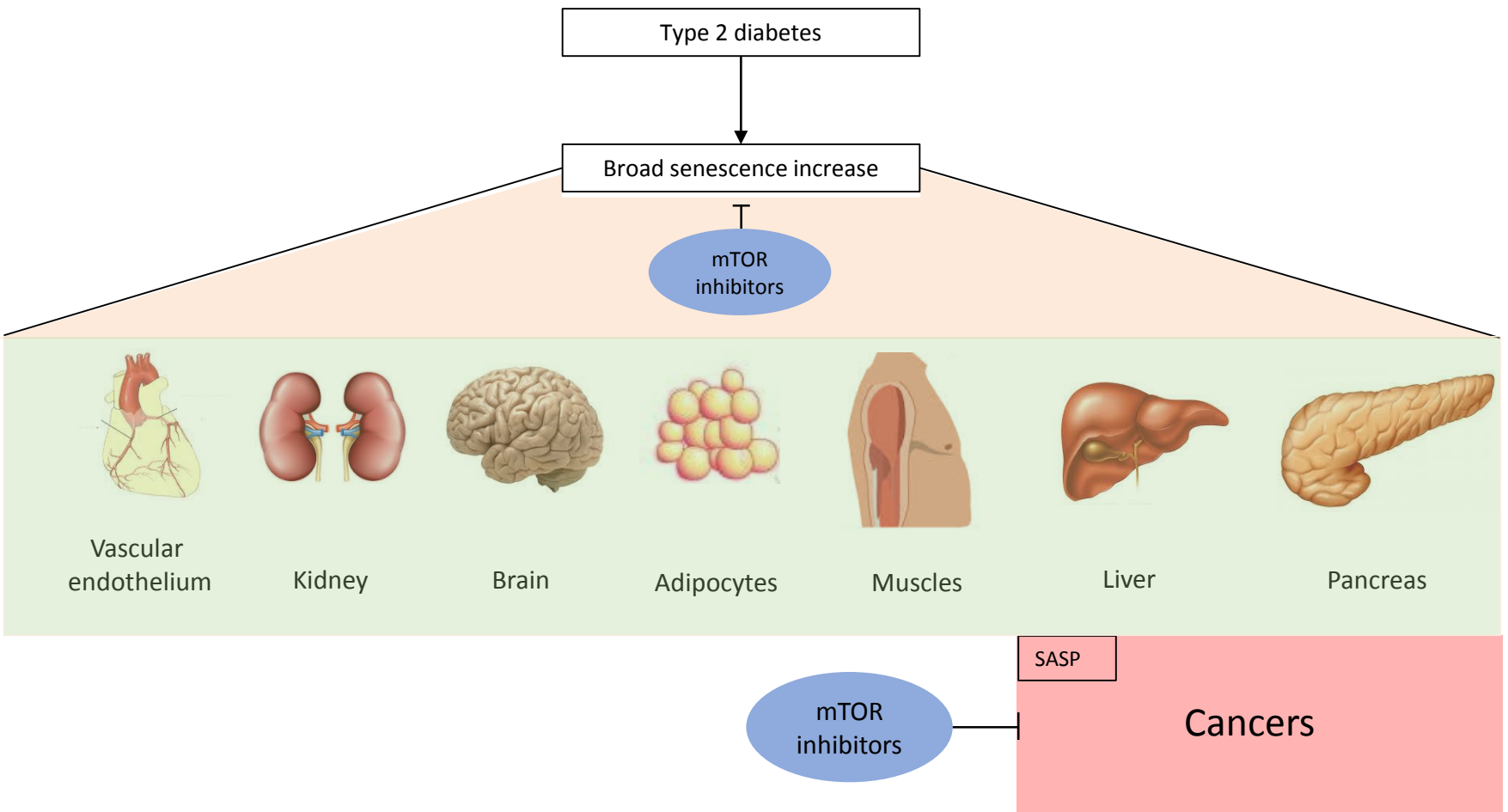

Supplement: Supplementary file 8 — Fig. S8 Comprehensive overview of the tissue specific impact of type 2 diabetes (T2DM) in the spectrum of the predictions from our model. [file ACEL-15-1018-s008.pdf]
