# Supplementary material for: A comprehensive approach to the molecular determinants of lifespan using a Boolean model of geroconversion
Source: Aging Cell. 2016 Sep 9;15(6):1018–26. doi: 10.1111/acel.12504 (PMC6398530; doi:10.1111/acel.12504)
Supplement: Supplementary file 6 — Fig. S6 mTOR inhibitors toxicity can be deduced from the estimated doubling times. [file ACEL-15-1018-s006.pdf]

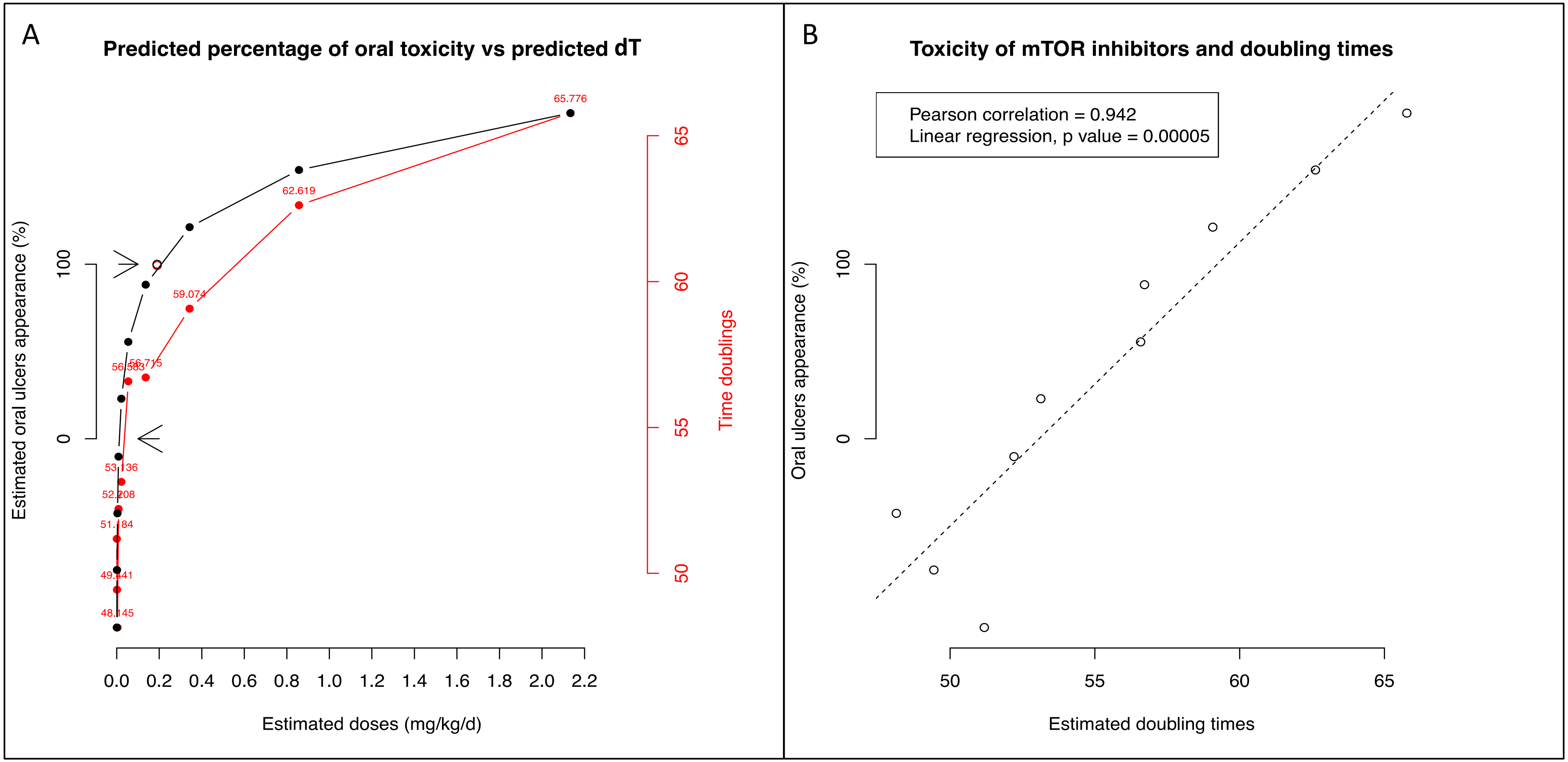

A
Predicted percentage of oral toxicity vs predicted dT
Estimated oral ulcers appearance (%)
100
0
65.776
62.619
59.074
56.583
56.715
53.136
52.208
51.184
49.441
48.145
65
60
55
50
Time doublings
0.0
0.2
0.4
0.6
0.8
1.0
1.2
1.4
1.6
1.8
2.0
2.2
Estimated doses (mg/kg/d)
B
Toxicity of mTOR inhibitors and doubling times
Pearson correlation = 0.942
Linear regression, p value = 0.00005
Oral ulcers appearance (%)
100
0
50
55
60
65
Estimated doubling times
