# Supplementary material for: A comprehensive approach to the molecular determinants of lifespan using a Boolean model of geroconversion
Source: Aging Cell. 2016 Sep 9;15(6):1018–26. doi: 10.1111/acel.12504 (PMC6398530; doi:10.1111/acel.12504)
Supplement: Supplementary file 9 — Table S1 List of literature references for the molecular network of geroconversion. Table S2 Details for the inhibitory effect of everolimus per dose in PBMC and pancreatic tumors. Table S3 Details for the effect of rapamycin dosage on liver degeneration in male mice. Table S4 Details for the pooled estimations of lifespan increase with rapamycin. Appendix S1 Constitutive regulations that can be found in the molecular network. Appendix S2 Rational for senescence studies selection. Appendix S3 Rational for cancer types & longevity studies selection. Appendix S4 MaBoSS script for the normal model presented in Fig 1 and containing the logical rules (without the T2DM feedback). [file ACEL-15-1018-s009.doc]

Supporting information for the paper: A comprehensive approach to the molecular determinants of lifespan using a Boolean model of geroconversion.

**Supporting Tables**

***Supporting table 1* *(referring to figure 1):***

List of literature references for the molecular network of geroconversion.

| Interaction | References |
| --- | --- |
| MAPK -> P16 | (Serrano et al. 1997) |
| MAPK -| TSC | (Rodriguez-Viciana et al. 1994) (Ma et al. 2005) |
| MAPK -> MYC | (Bretones et al. 2015) |
| MYC -> CDK2 | (Hydbring et al. 2010) |
| MYC -| MDM2 | (Bretones et al. 2015) |
| MYC -> PRC | (Bracken et al. 2003) |
| E2F1 -> MYC | (Bretones et al. 2015) |
| E2F1 -> MYC | (Bretones et al. 2015) |
| mTORC1 -> MYC | (Gera et al. 2004) |
| RB1 -> E2F11  E2F1 -| p16 | (Takahashi et al. 2006) (Medema et al. 1995) |
| CDK2 -| RB1 | (Aguda & Algar 2003) |
| E2F1 -> CDK2 | (Aguda & Algar 2003) |
| IRS -> AKT | (Laplante & Sabatini 2012) |
| PP2A -| AKT | (Seshacharyulu et al. 2013) |
| PP2A -| MAPK | (Junttila et al. 2008) |
| PPP1 -> Metabolism | <http://www.kegg.jp/kegg-bin/highlight_pathway?scale=1.0&map=map04910&keyword=PP1C> |
| AKT -> PPP1 | [http://www.kegg.jp/kegg-bin/highlight_pathway?scale=1.0&map=map04910&keyword=PP1C](http://www.kegg.jp/kegg-bin/highlight_pathway?scale=1.0&map=map04910&keyword=ppp1c) |
| CDK2 -> AKT | (Liu, Begley, et al. 2014) |
| AKT -| TSC  TSC -| mTORC1 | (Inoki et al. 2002) |
| mTORC1 -| MDM2 | (Lai et al. 2010) (Lee et al. 2007, p.53) |
| AKT -> MDM2 | (Mayo & Donner 2001) |
| mTORC1 -> CDK2 | (Jefferies et al. 1997) |
| mTORC1 -| PP2 | (Ma & Blenis 2009) |
| P16 -> MDM2 | (Mirzayans et al. 2012) |
| P53 -> PTEN | (Chang et al. 2008) |
| P53 -> AMPK | (Hasty et al. 2013) (Madan et al. 2011) |
| AMPK -> TSC | (Inoki et al. 2003) |
| ATP -| AMPK | (Hasty et al. 2013) (Hahn-Windgassen et al. 2005) |
| AKT -> Metabolism  mTORC1 -> Metabolism  Metabolism -> ATP | (Hahn-Windgassen et al. 2005) |
| AKT -| FOXO | (Guertin et al. 2006, p.1) |
| AKT -| p21 | (Zhou et al. 2001) (Nicholson & Anderson 2002) |
| MYC -| p21 | (Bretones et al. 2015) |
| mTORC1 -> Metabolism | (Ma & Blenis 2009) |
| AKT -| PRC | (Cha et al. 2005, p.3) |
| AMPK -> FOXO | (Greer et al. 2007) |
| Metabolism -> FOXO | (Jaeschke & Davis 2007) (Essers et al. 2004) |
| AKT -> PP1C  MAPK -> PP1C  PP1C -> Metabolism | Insulin signaling: <http://www.kegg.jp/kegg-bin/show_pathway?ko04910+K06269>  MAPK signaling: <http://lsresearch.thomsonreuters.com/static/maps/723_map.png> |
| mTORC1 -| PP2A | (Li et al. 2013) |
| PP2A -| MAPK  PP2A -| AKT | (Junttila et al. 2008) |
| PTEN -| AKT | (Leslie et al. 2009) |
| P16 -> Senescence | (Mirzayans et al. 2012) |
| P21 & mTORC1 -> Senescence | (Astle et al. 2012) (Feng & Levine 2010) (Blagosklonny 2014) (Hasty et al. 2013) |
| Metabolism | corresponds to: Glycolysis, glycogenesis and protein synthesis:  <http://www.kegg.jp/kegg-bin/highlight_pathway?scale=1.0&map=map04910&keyword=insulin> |
| MAPK | corresponds to: HER, RAS, RAF, MEK, ERK:  <http://www.kegg.jp/kegg-bin/show_pathway?map=hsa05200&show_description=show>  <http://www.kegg.jp/kegg-bin/show_pathway?map=hsa04010&show_description=show> |

***Supporting table 2 (referring to figure 5A and supporting figure 1):***

|  | Plot | Treatment dose (mg) | Transformation factor | Rate for the « Therapy » simulations | mTORC1_S6K1 inhibition at 24h |
| --- | --- | --- | --- | --- | --- |
| PBMC | A | 0.5 | 1 | X1=0.035 | 19% |
|  | *B* | *5* | *10* | *X1/10=0.0035* | *60%* |
|  | C | 10 | 20 | X1/20=0.00175 | 72% |
|  | D | 20 | 40 | X1/40=0.000875 | 79% |
|  | E | 30 | 60 | X1/60=0.0005833 | 82% |
|  | F | 50 | 100 | X1/100=0.00035 | 85% |
|  | G | 70 | 140 | X1/140=0.00025 | 86% |
| Pancreatic tumors | H | 0.5 | 1 | X1=0.035 | 0% |
|  | I | 5 | 10 | X1/10=0.0035 | 41% |
|  | J | 10 | 20 | X1/20=0.00175 | 55% |
|  | K | 20 | 40 | X1/40=0.000875 | 64% |
|  | L | 30 | 60 | X1/60=0.0005833 | 67% |
|  | M | 50 | 100 | X1/100=0.00035 | 70% |
|  | N | 70 | 140 | X1/140=0.00025 | 71% |

***Supporting table 3 (referring to figure 5B and supporting figure 2):***

| Plot | Treatment dose (mg/kg) | Rate for the « Therapy » simulations | Activity of the « Therapy » at 24h | Mean activity for daily administration |
| --- | --- | --- | --- | --- |
| A | 0.25 | 0.07 | 19% | 0.691 |
| B | 0.7466 | 0.0234959 | 57% | 0.787 |
| C | 2.24 | 0.0078125 | 83% | 0.916 |

***Supporting table 4 (referring to figure 5C and supporting figure 3 and 4):***

| Plot | Treatment dose (mg/kg) | Rate for the « Therapy » simulations | Activity of the « Therapy » at 24h | Mean activity for daily administration |
| --- | --- | --- | --- | --- |
| A | 2.24 | 0.007812 | 83% | 0.915 |
| B | 3.21428 | 0.00544425 | 88% | 0.939 |
| C | 0.4821428 | 0.0362963 | 42% | 0.711 |

**Supporting figures**

***Supporting figure 1***

Simulations of single doses of everolimus on PBMC and pancreatic tumors.

- The inhibition of S6K1 for a single 5 mg dose of everolimus reported in the literature for peripheral blood mononuclear cells (PBMC) has been used to calibrate the model to obtain a similar probability of mTORC1_S6K1 inhibition, represented by the *B)* raw in the table (Tanaka et al. 2008) (Boulay et al. 2004). We have therefore deduced the slope of decrease of the variable “Therapy” corresponding to 5mg (details concerning the syntax can be found in Material and methods).
- *A & C-N)* Other slopes of decrease of the variable “Therapy” have been reproduced by a proportional transformation of the rates calculated for B.
- For each simulation, the arbitrary time scale has been set to 168, corresponding to the 168 hours’ time point for the data in the literature. The mTORC1_S6K1 inhibition at 168 hours has been calculated by: 1-probabilities (mTORC1_S6K1).

***Supporting figure 2***

Simulations of single and daily administrations of rapamycin on the dose-related liver degeneration.

- A-C) The rates of decrease of the variable “Therapy” have been calculated as previously shown, considering 0.5 mg/kg as the calibrated dose from which the other rates are deduced.
- D-F) The mean activity for daily administration has been calculated by (1+ Activity of the « Therapy » at 24h)/2.

***Supporting figure 3***

Simulations of single and daily administrations of rapamycin on the dose-related lifespan expansion in mice.

- A-C) The rates of decrease of the variable “Therapy” have been calculated as previously shown, considering 0.5 mg/kg as the calibrated dose from which the other rates are deduced.
- For longevity study #1 the dose administered was 2.24 mg/kg/day.
- For longevity study #2 rapamycin was administered subcutaneously consisting on 6 injections at 1.5mg/kg in 4 weeks, which corresponds to (6*1.5)/(28*0.1) = 3.21428mg/kg/day.
- For longevity study #3 rapamycin was administered with subcutaneously consisting on 3 injections at 0.45mg/kg in 4 weeks which corresponds to (3*0.45)/(28*0.1) = 0.4821428mg/kg/day. The factor 0.1 represents the bioavability of the subcutaneous administration in comparison to the oral administration (Crowe et al. 1999).
- The mean activity for daily administration has been calculated by (1+ Activity of the « Therapy » at 24h)/2.

***Supporting figure 4***

Simulations of the survival increase with rapamycin.

- The experimental protocol corresponding to each longevity study has been simulated with and without rapamycin.
- The linear functions of senescence have been reported from 100% to 0% by the equation: f(t) = 1-Senescence*t.
- The difference in survival between the control and rapamycin simulations have been calculated at 90% mean mortality and, for longevity study #2, at 50% mean mortality.
- Comparison with the results of the 3 longevity studies are reported in *figure 6.*
- WT: wild type

***Supporting figure 5:***

Relation between rapamycin dose and toxicity.

- Published studies have been used to deduce a dose-related oral ulcers appearance for everolimus and rapamycin (Ferté et al. 2011) (Mahé et al. 2005) (MacDonald & RAPAMUNE Global Study Group 2001).
- The fitted linear regression model used in *Fig 7* is: f(log(Dose))= 159.51 + 35.74* log(Dose).

***Supporting figure 6:***

mTOR inhibitors toxicity can be deduced from the estimated doubling times.

- A) The estimations of the doubling times have been calculated from the simulations for each dose with the formula: dT= log(2)/( G1_S - G1_S*Senescence) (Material and methods). Arrows represent 0% and 100% oral ulcers appearance.
- B) The correlation between the doubling times and the oral ulcers appearance suggests the model precisely predicts toxicities of mTOR inhibitors.

***Supporting figure 7***:

The anti-proliferative effect of rapamycin.

- HER2-neu mice have been simulated by MAPK hyper activation.
- The doubling times (dT) have been calculated with the formula: dT= log(2)/( G1_S - G1_S*Senescence), and the functions of the curves: f(t) = exp(G1_S*t - G1_S*Senescence*t) (Material and methods).
- The % reduction corresponds to: 100*(105.462-138.94)/105.462 = -31.744.

***Supporting figure 8***:

Comprehensive overview of the tissue specific impact of type 2 diabetes (T2DM) in the spectrum of the predictions from our model.

- For vascular endothelium, kidney, brain, adipocytes and muscle tissues, the molecular network altered by T2DM increases cell senescence, and impact the tissue function.
- Cancers from the hepatobiliary tract are specifically overrepresented in T2DM patients compared to the population-wide distribution.

**Supporting informations**

***Supporting information 1 (referring to figure 1):***

Constitutive regulations that can be found in the molecular network.

Using GINsim software, we have analyzed the functional circuits in the normal model (Naldi et al. 2009, p.3). The negative functional circuits (corresponding to negative feedback loops) involve p53/PTEN and AMPK activities whereas the positive functional circuits involve the ATP load, CDK2 and PP2 activities. In addition, we can visually deduce from the model that MYC and p53 proteins have antagonistic influences on CDKN1A and MDM2 (Bretones et al. 2015) (el-Deiry et al. 1993). MYC and p53 proteins are also engaged in a functional circuit with CDK2 and AKT, enforcing the complex regulations between the cell cycling regulatory system, the PI3K/AKT/mTOR and the p53/CDKN1A pathways (Aguda & Algar 2003) (Liu, Begley, et al. 2014).

***Supporting information 2:***

Rational for senescence studies selection.

Many studies have evaluated the relation between metabolic diseases and cell senescence in distinct tissue types. To be compared with the model, studies required at least a quantification of cell senescence in the normal and diabetic situations. The rational to exclude studies from the analysis are appended to the description of every tissue type thereafter.

Nonalcoholic fatty liver disease (NAFLD)

T2DM is a well-known risk factor for NAFLD, the first cause of cirrhosis for nonalcoholic patients (Adams et al. 2005) (Eguchi et al. 2006) (Richardson et al. 2007). Interestingly, a telomere shortening-independent increase in cell senescence has been reported in NAFLD, constituting a prognostic marker of severity (Nakajima et al. 2010). Furthermore, T2DM is suspected to be causal for the hepatocyte’s senescence increase observed in NAFLD (Aravinthan et al. 2013) (Yang et al. 2004).

One study has not been used for comparison because the diabetic condition was assessed by HOMA-IR which is not transposable to our modeling approach (Richardson et al. 2007).

Vascular complications

Endothelial cells are relevant for the cardio-vascular complications that constitute the main cause of T2DM-related mortality. Increased cell senescence is suspected to be a key feature in the physiopathology of cardio-vascular complications of T2DM (Shinmura et al. 2011) (Wang et al. 2009) (Yuan et al. 2015). A single study related to the subject was not used because the diabetic model was questionable (Arunachalam et al. 2014, p.1).

Diabetic nephropathy

Diabetic nephropathy and peripheral aggravation of insulin resistance are also common complications of T2DM in the clinic (Verzola et al. 2008) (Liu, Huang, et al. 2014) (Minamino et al. 2009). Though reporting a 3-fold increase in senescence proportions, consistent with our model predictions, a study was not used for comparison because the senescence proportions in the normal and diabetic conditions were surprisingly low compared to similar studies (Verzola et al. 2008).

Dementia

The whole brain volume of T2DM patients shrinks 3 times faster than in normal aging (Biessels & Reijmer 2014). Indeed, the whole brain volume shrinkage is a hallmark of dementia such as Alzheimer disease, which is associated with T2DM (Luchsinger et al. 2007) (Zhao & Townsend 2009) (De Felice 2013) (Katon et al. 2012). Clinical studies used for comparison with the model are, to our knowledge, exhaustive. Although astrocyte senescence is a well-demonstrated pattern of Alzheimer disease, the link with T2DM and senescence is not frequently investigated (Simpson et al. 2010) (Pertusa et al. 2007) (Bhat et al. 2012) (Chinta et al. 2015). High neuroinflammation is extensively reported in dementia, related to IL-6 and IL-1 (Salminen et al. 2011) (Laberge et al. 2015). The secretion of IL-6 and IL-1 by senescent cells (SASP) further reinforces the link between T2DM, senescence and dementia. Moreover, the therapeutic inhibition of mTOR by rapamycin protects mice against atherosclerosis, cognition loss and Alzheimer disease (Flynn et al. 2013) (Spilman et al. 2010) (Halloran et al. 2012).

Pancreatic beta islet cells’ senescence

The number of pancreatic beta islet cells, responsible for insulin secretion, decreases during aging and/or metabolic diseases, a process responsible of the amplification of peripheral hyperglucosemia (Gargani et al. 2013) (Teta et al. 2005) (Reers et al. 2009) (Dalbøge et al. 2013). To our knowledge, a single study has reported a 4.7-fold increase in beta-galactosidase-positive (i.e.: senescent) beta islet area for mice with high fat diet (Sone & Kagawa 2005). This effect is close to the difference reported in our simulations, however with lower initial proportions of senescent cells compared to other biological data fit by our model. Importantly, beta islet cells senescence is responsible of the amplification of peripheral hyperglucosemia and T2DM (Muñoz-Espín & Serrano 2014).

***Supporting information 3:***

Rational for cancer types & longevity studies selection.

T2DM and cancer types

Cancer types related to T2DM are strikingly unusual. Hepatocarcinomas, cholangiocarcinomas and pancreatic cancers (collectively referred to as cancers affecting the hepatobiliary tract) are specifically overrepresented in T2DM patients compared to the population-wide distribution (Tsilidis et al. 2015). Indeed, we have already pointed out the role of cell senescence in NAFLD and fibrosis development, ultimately leading to cirrhosis, which in turn is the main risk-factor for both hepatocarcinoma and cholangiocarcinoma (Palmer & Patel 2012) (Llovet et al. 2015). Interestingly, IL-6, which as noted above is secreted by senescent cells, plays a central role in cholangiocarcinoma’s formation (Wehbe et al. 2006) (Laberge et al. 2015). On the other hand, a permissive microenvironment also greatly promotes pancreatic cancer development, in which IL-6 seems to be also a key player (Zhang et al. 2013). Moreover, resistance to senescence is a decisive step in hepatocarcinoma formation (Schulze et al. 2015).

Longevity studies selection

We selected longevity studies that compared survival under rapamycin versus no treatment in large mice cohorts, with details concerning dose administration and survival parameters. Dietary restriction studies were excluded because we couldn’t extrapolate its effect on the percentage of inhibition of a node in our model. The rational to exclude studies that approximated the selection criteria are the following: The study of Beadling, Neff and colleagues didn’t provide the raw data for survival (Beadling et al. 2013). The study of Chen and colleagues was performed with intraperitoneal injection of rapamycin, for which no information on treatment pharmacodynamics is provided (Chen et al. 2009). Although properly conducted, the study of Leontieva and colleagues has used an obese mice model, multiple doses in small groups, and mice were sacrificed after one year of treatment, leading to numerous uncertainties preventing fair comparison with our model (Leontieva et al. 2014).

***Supporting information 4*:**

MaBoSS script for the normal model presented in *Fig 1* and containing the logical rules (without the T2DM feedback).

File .bnd:

Node Insulin {

logic = (Insulin);

rate_up = @logic ? $u_Insulin : 0;

rate_down = @logic ? 0 : $d_Insulin;

}

Node GF {

logic = (GF);

rate_up = @logic ? $u_GF : 0;

rate_down = @logic ? 0 : $d_GF;

}

Node Senescence {

logic = (!p16 & p21 & mTORC1_S6K1) | (p16);

rate_up = @logic ? $u_Senescence : 0;

rate_down = @logic ? 0 : $d_Senescence;

}

Node G1_S {

logic = (!p21 & CDK2 & E2F1 & Metabolism);

rate_up = @logic ? $u_G1_S : 0;

rate_down = @logic ? 0 : $d_G1_S;

}

Node MAPK {

logic = (GF & !PP2A);

rate_up = @logic ? $u_MAPK : 0;

rate_down = @logic ? 0 : $d_MAPK;

}

Node p16 {

logic = (MAPK & !p53 & !E2F1 & !PRC);

rate_up = @logic ? $u_p16 : 0;

rate_down = @logic ? 0 : $d_p16;

}

Node MDM2 {

logic = (!p16 & !p53 & AKT & !mTORC1_S6K1 & !MYC & !E2F1) | (!p16 & p53 & !mTORC1_S6K1 & !MYC & !E2F1) | (p16 & !mTORC1_S6K1 & !MYC & !E2F1);

rate_up = @logic ? $u_MDM2 : 0;

rate_down = @logic ? 0 : $d_MDM2;

}

Node p53 {

logic = (!MDM2);

rate_up = @logic ? $u_p53 : 0;

rate_down = @logic ? 0 : $d_p53;

}

Node p21 {

logic = (!p53 & !AKT & !MYC & FOXO) | (p53 & !AKT & !MYC);

rate_up = @logic ? $u_p21 : 0;

rate_down = @logic ? 0 : $d_p21;

}

Node AKT {

logic = (!IRS_PIK3CA & !PTEN & CDK2 & !PP2A) | (IRS_PIK3CA & !PTEN & !PP2A);

rate_up = @logic ? $u_AKT : 0;

rate_down = @logic ? 0 : $d_AKT;

}

Node mTORC1_S6K1 {

logic = (!AMPK & !TSC);

rate_up = @logic ? $u_mTORC1_S6K1 : 0;

rate_down = @logic ? 0 : $d_mTORC1_S6K1;

}

Node ATP {

logic = (Metabolism);

rate_up = @logic ? $u_ATP : 0;

rate_down = @logic ? 0 : $d_ATP;

}

Node IRS_PIK3CA {

logic = (Insulin);

rate_up = @logic ? $u_IRS_PIK3CA : 0;

rate_down = @logic ? 0 : $d_IRS_PIK3CA;

}

Node AMPK {

logic = (p53 & !ATP);

rate_up = @logic ? $u_AMPK : 0;

rate_down = @logic ? 0 : $d_AMPK;

}

Node PTEN {

logic = (p53 & !AKT);

rate_up = @logic ? $u_PTEN : 0;

rate_down = @logic ? 0 : $d_PTEN;

}

Node TSC {

logic = (!MAPK & !AKT & AMPK);

rate_up = @logic ? $u_TSC : 0;

rate_down = @logic ? 0 : $d_TSC;

}

Node MYC {

logic = (MAPK & !p53 & mTORC1_S6K1 & E2F1);

rate_up = @logic ? $u_MYC : 0;

rate_down = @logic ? 0 : $d_MYC;

}

Node CDK2 {

logic = (!p21 & mTORC1_S6K1 & !MYC & E2F1) | (!p21 & mTORC1_S6K1 & MYC);

rate_up = @logic ? $u_CDK2 : 0;

rate_down = @logic ? 0 : $d_CDK2;

}

Node RB1 {

logic = (!CDK2);

rate_up = @logic ? $u_RB1 : 0;

rate_down = @logic ? 0 : $d_RB1;

}

Node E2F1 {

logic = (!GF & MYC & !RB1 & E2F1) | (GF & !RB1 & E2F1);

rate_up = @logic ? $u_E2F1 : 0;

rate_down = @logic ? 0 : $d_E2F1;

}

Node PRC {

logic = (!AKT & MYC);

rate_up = @logic ? $u_PRC : 0;

rate_down = @logic ? 0 : $d_PRC;

}

Node Metabolism {

logic = (!MAPK & !AKT & mTORC1_S6K1 & PP1C) | (!MAPK & AKT & mTORC1_S6K1) | (MAPK & !AKT & PP1C) | (MAPK & AKT);

rate_up = @logic ? $u_Metabolism : 0;

rate_down = @logic ? 0 : $d_Metabolism;

}

Node PP2A {

logic = (!mTORC1_S6K1);

rate_up = @logic ? $u_PP2A : 0;

rate_down = @logic ? 0 : $d_PP2A;

}

Node FOXO {

logic = (!MAPK & !p16 & !AKT & !AMPK & Metabolism) | (!MAPK & !p16 & !AKT & AMPK) | (!MAPK & p16 & !AKT);

rate_up = @logic ? $u_FOXO : 0;

rate_down = @logic ? 0 : $d_FOXO;

}

Node PP1C {

logic = (!MAPK & AKT) | (MAPK);

rate_up = @logic ? $u_PP1C : 0;

rate_down = @logic ? 0 : $d_PP1C;

}

File .cfg:

$u_Insulin=1;

$d_Insulin=1;

$u_GF=1;

$d_GF=1;

$u_Senescence=1;

$d_Senescence=1;

$u_G1_S=1;

$d_G1_S=1;

$u_MAPK=1;

$d_MAPK=1;

$u_p16=1;

$d_p16=1;

$u_MDM2=1;

$d_MDM2=1;

$u_p53=1;

$d_p53=1;

$u_p21=1;

$d_p21=1;

$u_AKT=1;

$d_AKT=1;

$u_mTORC1_S6K1=1;

$d_mTORC1_S6K1=1;

$u_ATP=1;

$d_ATP=1;

$u_IRS_PIK3CA=1;

$d_IRS_PIK3CA=1;

$u_AMPK=1;

$d_AMPK=1;

$u_PTEN=1;

$d_PTEN=1;

$u_TSC=1;

$d_TSC=1;

$u_MYC=1;

$d_MYC=1;

$u_CDK2=1;

$d_CDK2=1;

$u_RB1=1;

$d_RB1=1;

$u_E2F1=1;

$d_E2F1=1;

$u_PRC=1;

$d_PRC=1;

$u_Metabolism=1;

$d_Metabolism=1;

$u_PP2A=1;

$d_PP2A=1;

$u_FOXO=1;

$d_FOXO=1;

$u_PP1C=1;

$d_PP1C=1;

Insulin.is_internal=1;

GF.is_internal=1;

Senescence.is_internal=0;

G1_S.is_internal=0;

MAPK.is_internal=1;

p16.is_internal=1;

MDM2.is_internal=1;

p53.is_internal=1;

p21.is_internal=1;

AKT.is_internal=1;

mTORC1_S6K1.is_internal=1;

ATP.is_internal=1;

IRS_PIK3CA.is_internal=1;

AMPK.is_internal=1;

PTEN.is_internal=1;

TSC.is_internal=1;

MYC.is_internal=1;

CDK2.is_internal=1;

RB1.is_internal=1;

E2F1.is_internal=1;

PRC.is_internal=1;

Metabolism.is_internal=1;

PP2A.is_internal=1;

FOXO.is_internal=1;

PP1C.is_internal=1;

discrete_time = 0;

use_physrandgen = FALSE;

seed_pseudorandom = 100;

sample_count = 50000;

max_time = 400;

time_tick = 0.1;

thread_count = 4;

statdist_traj_count = 100;

statdist_cluster_threshold = 0.9;

**References:**

Adams LA, Sanderson S, Lindor KD & Angulo P (2005) The histological course of nonalcoholic fatty liver disease: a longitudinal study of 103 patients with sequential liver biopsies. *J. Hepatol.* 42, 132–138.

Aguda BD & Algar CK (2003) A structural analysis of the qualitative networks regulating the cell cycle and apoptosis. *Cell Cycle* 2, 538–544.

Aravinthan A, Scarpini C, Tachtatzis P, Verma S, Penrhyn-Lowe S, Harvey R, Davies SE, Allison M, Coleman N & Alexander G (2013) Hepatocyte senescence predicts progression in non-alcohol-related fatty liver disease. *J. Hepatol.* 58, 549–556.

Astle MV, Hannan KM, Ng PY, Lee RS, George AJ, Hsu AK, Haupt Y, Hannan RD & Pearson RB (2012) AKT induces senescence in human cells via mTORC1 and p53 in the absence of DNA damage: implications for targeting mTOR during malignancy. *Oncogene* 31, 1949–1962.

Beadling C, Neff TL, Heinrich MC, Rhodes K, Thornton M, Leamon J, Andersen M & Corless CL (2013) Combining highly multiplexed PCR with semiconductor-based sequencing for rapid cancer genotyping. *J Mol Diagn* 15, 171–176.

Bhat R, Crowe EP, Bitto A, Moh M, Katsetos CD, Garcia FU, Johnson FB, Trojanowski JQ, Sell C & Torres C (2012) Astrocyte senescence as a component of Alzheimer’s disease. *PLoS ONE* 7, e45069.

Biessels GJ & Reijmer YD (2014) Brain changes underlying cognitive dysfunction in diabetes: what can we learn from MRI? *Diabetes* 63, 2244–2252.

Blagosklonny MV (2014) Geroconversion: irreversible step to cellular senescence. *Cell Cycle* 13, 3628–3635.

Boulay A, Zumstein-Mecker S, Stephan C, Beuvink I, Zilbermann F, Haller R, Tobler S, Heusser C, O’Reilly T, Stolz B, Marti A, Thomas G & Lane HA (2004) Antitumor efficacy of intermittent treatment schedules with the rapamycin derivative RAD001 correlates with prolonged inactivation of ribosomal protein S6 kinase 1 in peripheral blood mononuclear cells. *Cancer Res.* 64, 252–261.

Bracken AP, Pasini D, Capra M, Prosperini E, Colli E & Helin K (2003) EZH2 is downstream of the pRB-E2F pathway, essential for proliferation and amplified in cancer. *EMBO J.* 22, 5323–5335.

Bretones G, Delgado MD & León J (2015) Myc and cell cycle control. *Biochim. Biophys. Acta* 1849, 506–516.

Chang C-J, Mulholland DJ, Valamehr B, Mosessian S, Sellers WR & Wu H (2008) PTEN nuclear localization is regulated by oxidative stress and mediates p53-dependent tumor suppression. *Mol. Cell. Biol.* 28, 3281–3289.

Cha T-L, Zhou BP, Xia W, Wu Y, Yang C-C, Chen C-T, Ping B, Otte AP & Hung M-C (2005) Akt-mediated phosphorylation of EZH2 suppresses methylation of lysine 27 in histone H3. *Science* 310, 306–310.

Chen C, Liu Y, Liu Y & Zheng P (2009) mTOR regulation and therapeutic rejuvenation of aging hematopoietic stem cells. *Sci Signal* 2, ra75.

Chinta SJ, Woods G, Rane A, Demaria M, Campisi J & Andersen JK (2015) Cellular senescence and the aging brain. *Exp. Gerontol.* 68, 3–7.

Crowe A, Bruelisauer A, Duerr L, Guntz P & Lemaire M (1999) Absorption and intestinal metabolism of SDZ-RAD and rapamycin in rats. *Drug Metab. Dispos.* 27, 627–632.

Dalbøge LS, Almholt DLC, Neerup TSR, Vassiliadis E, Vrang N, Pedersen L, Fosgerau K & Jelsing J (2013) Characterisation of age-dependent beta cell dynamics in the male db/db mice. *PLoS ONE* 8, e82813.

De Felice FG (2013) Alzheimer’s disease and insulin resistance: translating basic science into clinical applications. *J. Clin. Invest.* 123, 531–539.

el-Deiry WS, Tokino T, Velculescu VE, Levy DB, Parsons R, Trent JM, Lin D, Mercer WE, Kinzler KW & Vogelstein B (1993) WAF1, a potential mediator of p53 tumor suppression. *Cell* 75, 817–825.

Eguchi Y, Eguchi T, Mizuta T, Ide Y, Yasutake T, Iwakiri R, Hisatomi A, Ozaki I, Yamamoto K, Kitajima Y, Kawaguchi Y, Kuroki S & Ono N (2006) Visceral fat accumulation and insulin resistance are important factors in nonalcoholic fatty liver disease. *J. Gastroenterol.* 41, 462–469.

Essers MAG, Weijzen S, de Vries-Smits AMM, Saarloos I, de Ruiter ND, Bos JL & Burgering BMT (2004) FOXO transcription factor activation by oxidative stress mediated by the small GTPase Ral and JNK. *EMBO J.* 23, 4802–4812.

Feng Z & Levine AJ (2010) The regulation of energy metabolism and the IGF-1/mTOR pathways by the p53 protein. *Trends Cell Biol.* 20, 427–434.

Ferté C, Paci A, Zizi M, Gonzales DB, Goubar A, Gomez-Roca C, Massard C, Sahmoud T, André F & Soria J-C (2011) Natural history, management and pharmacokinetics of everolimus-induced-oral ulcers: insights into compliance issues. *Eur. J. Cancer* 47, 2249–2255.

Flynn JM, O’Leary MN, Zambataro CA, Academia EC, Presley MP, Garrett BJ, Zykovich A, Mooney SD, Strong R, Rosen CJ, Kapahi P, Nelson MD, Kennedy BK & Melov S (2013) Late-life rapamycin treatment reverses age-related heart dysfunction. *Aging Cell* 12, 851–862.

Gargani S, Thévenet J, Yuan JE, Lefebvre B, Delalleau N, Gmyr V, Hubert T, Duhamel A, Pattou F & Kerr-Conte J (2013) Adaptive changes of human islets to an obesogenic environment in the mouse. *Diabetologia* 56, 350–358.

Gera JF, Mellinghoff IK, Shi Y, Rettig MB, Tran C, Hsu J, Sawyers CL & Lichtenstein AK (2004) AKT activity determines sensitivity to mammalian target of rapamycin (mTOR) inhibitors by regulating cyclin D1 and c-myc expression. *J. Biol. Chem.* 279, 2737–2746.

Greer EL, Oskoui PR, Banko MR, Maniar JM, Gygi MP, Gygi SP & Brunet A (2007) The energy sensor AMP-activated protein kinase directly regulates the mammalian FOXO3 transcription factor. *J. Biol. Chem.* 282, 30107–30119.

Guertin DA, Stevens DM, Thoreen CC, Burds AA, Kalaany NY, Moffat J, Brown M, Fitzgerald KJ & Sabatini DM (2006) Ablation in mice of the mTORC components raptor, rictor, or mLST8 reveals that mTORC2 is required for signaling to Akt-FOXO and PKCalpha, but not S6K1. *Dev. Cell* 11, 859–871.

Hahn-Windgassen A, Nogueira V, Chen C-C, Skeen JE, Sonenberg N & Hay N (2005) Akt activates the mammalian target of rapamycin by regulating cellular ATP level and AMPK activity. *J. Biol. Chem.* 280, 32081–32089.

Halloran J, Hussong SA, Burbank R, Podlutskaya N, Fischer KE, Sloane LB, Austad SN, Strong R, Richardson A, Hart MJ & Galvan V (2012) Chronic inhibition of mammalian target of rapamycin by rapamycin modulates cognitive and non-cognitive components of behavior throughout lifespan in mice. *Neuroscience* 223, 102–113.

Hasty P, Sharp ZD, Curiel TJ & Campisi J (2013) mTORC1 and p53: clash of the gods? *Cell Cycle* 12, 20–25.

Hydbring P, Bahram F, Su Y, Tronnersjö S, Högstrand K, von der Lehr N, Sharifi HR, Lilischkis R, Hein N, Wu S, Vervoorts J, Henriksson M, Grandien A, Lüscher B & Larsson L-G (2010) Phosphorylation by Cdk2 is required for Myc to repress Ras-induced senescence in cotransformation. *Proc. Natl. Acad. Sci. U.S.A.* 107, 58–63.

Inoki K, Li Y, Zhu T, Wu J & Guan K-L (2002) TSC2 is phosphorylated and inhibited by Akt and suppresses mTOR signalling. *Nat. Cell Biol.* 4, 648–657.

Inoki K, Zhu T & Guan K-L (2003) TSC2 mediates cellular energy response to control cell growth and survival. *Cell* 115, 577–590.

Jaeschke A & Davis RJ (2007) Metabolic stress signaling mediated by mixed-lineage kinases. *Mol. Cell* 27, 498–508.

Jefferies HB, Fumagalli S, Dennis PB, Reinhard C, Pearson RB & Thomas G (1997) Rapamycin suppresses 5’TOP mRNA translation through inhibition of p70s6k. *EMBO J.* 16, 3693–3704.

Junttila MR, Li S-P & Westermarck J (2008) Phosphatase-mediated crosstalk between MAPK signaling pathways in the regulation of cell survival. *FASEB J.* 22, 954–965.

Katon W, Lyles CR, Parker MM, Karter AJ, Huang ES & Whitmer RA (2012) Association of depression with increased risk of dementia in patients with type 2 diabetes: the Diabetes and Aging Study. *Arch. Gen. Psychiatry* 69, 410–417.

Laberge R-M, Sun Y, Orjalo AV, Patil CK, Freund A, Zhou L, Curran SC, Davalos AR, Wilson-Edell KA, Liu S, Limbad C, Demaria M, Li P, Hubbard GB, Ikeno Y, Javors M, Desprez P-Y, Benz CC, Kapahi P, Nelson PS & Campisi J (2015) MTOR regulates the pro-tumorigenic senescence-associated secretory phenotype by promoting IL1A translation. *Nat. Cell Biol.* 17, 1049–1061.

Lai KP, Leong WF, Chau JFL, Jia D, Zeng L, Liu H, He L, Hao A, Zhang H, Meek D, Velagapudi C, Habib SL & Li B (2010) S6K1 is a multifaceted regulator of Mdm2 that connects nutrient status and DNA damage response. *EMBO J.* 29, 2994–3006.

Laplante M & Sabatini DM (2012) mTOR signaling in growth control and disease. *Cell* 149, 274–293.

Lee C-H, Inoki K, Karbowniczek M, Petroulakis E, Sonenberg N, Henske EP & Guan K-L (2007) Constitutive mTOR activation in TSC mutants sensitizes cells to energy starvation and genomic damage via p53. *EMBO J.* 26, 4812–4823.

Leontieva OV, Paszkiewicz GM & Blagosklonny MV (2014) Weekly administration of rapamycin improves survival and biomarkers in obese male mice on high-fat diet. *Aging Cell* 13, 616–622.

Leslie NR, Maccario H, Spinelli L & Davidson L (2009) The significance of PTEN’s protein phosphatase activity. *Adv. Enzyme Regul.* 49, 190–196.

Liu J, Huang K, Cai G-Y, Chen X-M, Yang J-R, Lin L-R, Yang J, Huo B-G, Zhan J & He Y-N (2014) Receptor for advanced glycation end-products promotes premature senescence of proximal tubular epithelial cells via activation of endoplasmic reticulum stress-dependent p21 signaling. *Cell. Signal.* 26, 110–121.

Liu P, Begley M, Michowski W, Inuzuka H, Ginzberg M, Gao D, Tsou P, Gan W, Papa A, Kim BM, Wan L, Singh A, Zhai B, Yuan M, Wang Z, Gygi SP, Lee TH, Lu K-P, Toker A, Pandolfi PP, Asara JM, Kirschner MW, Sicinski P, Cantley L & Wei W (2014) Cell-cycle-regulated activation of Akt kinase by phosphorylation at its carboxyl terminus. *Nature* 508, 541–545.

Li Y, Wang X, Yue P, Tao H, Ramalingam SS, Owonikoko TK, Deng X, Wang Y, Fu H, Khuri FR & Sun S-Y (2013) Protein phosphatase 2A and DNA-dependent protein kinase are involved in mediating rapamycin-induced Akt phosphorylation. *J. Biol. Chem.* 288, 13215–13224.

Llovet JM, Villanueva A, Lachenmayer A & Finn RS (2015) Advances in targeted therapies for hepatocellular carcinoma in the genomic era. *Nat Rev Clin Oncol*.

Luchsinger JA, Reitz C, Patel B, Tang M-X, Manly JJ & Mayeux R (2007) Relation of diabetes to mild cognitive impairment. *Arch. Neurol.* 64, 570–575.

MacDonald AS & RAPAMUNE Global Study Group (2001) A worldwide, phase III, randomized, controlled, safety and efficacy study of a sirolimus/cyclosporine regimen for prevention of acute rejection in recipients of primary mismatched renal allografts. *Transplantation* 71, 271–280.

Madan E, Gogna R, Bhatt M, Pati U, Kuppusamy P & Mahdi AA (2011) Regulation of glucose metabolism by p53: emerging new roles for the tumor suppressor. *Oncotarget* 2, 948–957.

Mahé E, Morelon E, Lechaton S, Sang K-HLQ, Mansouri R, Ducasse M-F, Mamzer-Bruneel M-F, de Prost Y, Kreis H & Bodemer C (2005) Cutaneous adverse events in renal transplant recipients receiving sirolimus-based therapy. *Transplantation* 79, 476–482.

Ma L, Chen Z, Erdjument-Bromage H, Tempst P & Pandolfi PP (2005) Phosphorylation and functional inactivation of TSC2 by Erk implications for tuberous sclerosis and cancer pathogenesis. *Cell* 121, 179–193.

Ma XM & Blenis J (2009) Molecular mechanisms of mTOR-mediated translational control. *Nat. Rev. Mol. Cell Biol.* 10, 307–318.

Mayo LD & Donner DB (2001) A phosphatidylinositol 3-kinase/Akt pathway promotes translocation of Mdm2 from the cytoplasm to the nucleus. *Proc. Natl. Acad. Sci. U.S.A.* 98, 11598–11603.

Medema RH, Herrera RE, Lam F & Weinberg RA (1995) Growth suppression by p16ink4 requires functional retinoblastoma protein. *Proc. Natl. Acad. Sci. U.S.A.* 92, 6289–6293.

Minamino T, Orimo M, Shimizu I, Kunieda T, Yokoyama M, Ito T, Nojima A, Nabetani A, Oike Y, Matsubara H, Ishikawa F & Komuro I (2009) A crucial role for adipose tissue p53 in the regulation of insulin resistance. *Nat. Med.* 15, 1082–1087.

Mirzayans R, Andrais B, Hansen G & Murray D (2012) Role of p16(INK4A) in Replicative Senescence and DNA Damage-Induced Premature Senescence in p53-Deficient Human Cells. *Biochem Res Int* 2012, 951574.

Muñoz-Espín D & Serrano M (2014) Cellular senescence: from physiology to pathology. *Nat. Rev. Mol. Cell Biol.* 15, 482–496.

Nakajima T, Nakashima T, Okada Y, Jo M, Nishikawa T, Mitsumoto Y, Katagishi T, Kimura H, Itoh Y, Kagawa K & Yoshikawa T (2010) Nuclear size measurement is a simple method for the assessment of hepatocellular aging in non-alcoholic fatty liver disease: Comparison with telomere-specific quantitative FISH and p21 immunohistochemistry. *Pathol. Int.* 60, 175–183.

Naldi A, Berenguier D, Fauré A, Lopez F, Thieffry D & Chaouiya C (2009) Logical modelling of regulatory networks with GINsim 2.3. *BioSystems* 97, 134–139.

Nicholson KM & Anderson NG (2002) The protein kinase B/Akt signalling pathway in human malignancy. *Cell. Signal.* 14, 381–395.

Palmer WC & Patel T (2012) Are common factors involved in the pathogenesis of primary liver cancers? A meta-analysis of risk factors for intrahepatic cholangiocarcinoma. *J. Hepatol.* 57, 69–76.

Pertusa M, García-Matas S, Rodríguez-Farré E, Sanfeliu C & Cristòfol R (2007) Astrocytes aged in vitro show a decreased neuroprotective capacity. *J. Neurochem.* 101, 794–805.

Reers C, Erbel S, Esposito I, Schmied B, Büchler MW, Nawroth PP & Ritzel RA (2009) Impaired islet turnover in human donor pancreata with aging. *Eur. J. Endocrinol.* 160, 185–191.

Richardson MM, Jonsson JR, Powell EE, Brunt EM, Neuschwander-Tetri BA, Bhathal PS, Dixon JB, Weltman MD, Tilg H, Moschen AR, Purdie DM, Demetris AJ & Clouston AD (2007) Progressive fibrosis in nonalcoholic steatohepatitis: association with altered regeneration and a ductular reaction. *Gastroenterology* 133, 80–90.

Rodriguez-Viciana P, Warne PH, Dhand R, Vanhaesebroeck B, Gout I, Fry MJ, Waterfield MD & Downward J (1994) Phosphatidylinositol-3-OH kinase as a direct target of Ras. *Nature* 370, 527–532.

Schulze K, Imbeaud S, Letouzé E, Alexandrov LB, Calderaro J, Rebouissou S, Couchy G, Meiller C, Shinde J, Soysouvanh F, Calatayud A-L, Pinyol R, Pelletier L, Balabaud C, Laurent A, Blanc J-F, Mazzaferro V, Calvo F, Villanueva A, Nault J-C, Bioulac-Sage P, Stratton MR, Llovet JM & Zucman-Rossi J (2015) Exome sequencing of hepatocellular carcinomas identifies new mutational signatures and potential therapeutic targets. *Nat. Genet.* 47, 505–511.

Serrano M, Lin AW, McCurrach ME, Beach D & Lowe SW (1997) Oncogenic ras provokes premature cell senescence associated with accumulation of p53 and p16INK4a. *Cell* 88, 593–602.

Seshacharyulu P, Pandey P, Datta K & Batra SK (2013) Phosphatase: PP2A structural importance, regulation and its aberrant expression in cancer. *Cancer Lett.* 335, 9–18.

Shinmura K, Tamaki K, Sano M, Murata M, Yamakawa H, Ishida H & Fukuda K (2011) Impact of long-term caloric restriction on cardiac senescence: caloric restriction ameliorates cardiac diastolic dysfunction associated with aging. *J. Mol. Cell. Cardiol.* 50, 117–127.

Simpson JE, Ince PG, Haynes LJ, Theaker R, Gelsthorpe C, Baxter L, Forster G, Lace GL, Shaw PJ, Matthews FE, Savva GM, Brayne C, Wharton SB & MRC Cognitive Function and Ageing Neuropathology Study Group (2010) Population variation in oxidative stress and astrocyte DNA damage in relation to Alzheimer-type pathology in the ageing brain. *Neuropathol. Appl. Neurobiol.* 36, 25–40.

Sone H & Kagawa Y (2005) Pancreatic beta cell senescence contributes to the pathogenesis of type 2 diabetes in high-fat diet-induced diabetic mice. *Diabetologia* 48, 58–67.

Spilman P, Podlutskaya N, Hart MJ, Debnath J, Gorostiza O, Bredesen D, Richardson A, Strong R & Galvan V (2010) Inhibition of mTOR by rapamycin abolishes cognitive deficits and reduces amyloid-beta levels in a mouse model of Alzheimer’s disease. *PLoS ONE* 5, e9979.

Takahashi A, Ohtani N, Yamakoshi K, Iida S, Tahara H, Nakayama K, Nakayama KI, Ide T, Saya H & Hara E (2006) Mitogenic signalling and the p16INK4a-Rb pathway cooperate to enforce irreversible cellular senescence. *Nat. Cell Biol.* 8, 1291–1297.

Tanaka C, O’Reilly T, Kovarik JM, Shand N, Hazell K, Judson I, Raymond E, Zumstein-Mecker S, Stephan C, Boulay A, Hattenberger M, Thomas G & Lane HA (2008) Identifying optimal biologic doses of everolimus (RAD001) in patients with cancer based on the modeling of preclinical and clinical pharmacokinetic and pharmacodynamic data. *J. Clin. Oncol.* 26, 1596–1602.

Teta M, Long SY, Wartschow LM, Rankin MM & Kushner JA (2005) Very slow turnover of beta-cells in aged adult mice. *Diabetes* 54, 2557–2567.

Tsilidis KK, Kasimis JC, Lopez DS, Ntzani EE & Ioannidis JPA (2015) Type 2 diabetes and cancer: umbrella review of meta-analyses of observational studies. *BMJ* 350, g7607.

Verzola D, Gandolfo MT, Gaetani G, Ferraris A, Mangerini R, Ferrario F, Villaggio B, Gianiorio F, Tosetti F, Weiss U, Traverso P, Mji M, Deferrari G & Garibotto G (2008) Accelerated senescence in the kidneys of patients with type 2 diabetic nephropathy. *Am. J. Physiol. Renal Physiol.* 295, F1563–1573.

Wang C-Y, Kim H-H, Hiroi Y, Sawada N, Salomone S, Benjamin LE, Walsh K, Moskowitz MA & Liao JK (2009) Obesity increases vascular senescence and susceptibility to ischemic injury through chronic activation of Akt and mTOR. *Sci Signal* 2, ra11.

Wehbe H, Henson R, Meng F, Mize-Berge J & Patel T (2006) Interleukin-6 contributes to growth in cholangiocarcinoma cells by aberrant promoter methylation and gene expression. *Cancer Res.* 66, 10517–10524.

Yang S, Koteish A, Lin H, Huang J, Roskams T, Dawson V & Diehl AM (2004) Oval cells compensate for damage and replicative senescence of mature hepatocytes in mice with fatty liver disease. *Hepatology* 39, 403–411.

Yuan Q, Hu C-P, Gong Z-C, Bai Y-P, Liu S-Y, Li Y-J & Jiang J-L (2015) Accelerated onset of senescence of endothelial progenitor cells in patients with type 2 diabetes mellitus: role of dimethylarginine dimethylaminohydrolase 2 and asymmetric dimethylarginine. *Biochem. Biophys. Res. Commun.* 458, 869–876.

Zhang Y, Yan W, Collins MA, Bednar F, Rakshit S, Zetter BR, Stanger BZ, Chung I, Rhim AD & di Magliano MP (2013) Interleukin-6 is required for pancreatic cancer progression by promoting MAPK signaling activation and oxidative stress resistance. *Cancer Res.* 73, 6359–6374.

Zhao W-Q & Townsend M (2009) Insulin resistance and amyloidogenesis as common molecular foundation for type 2 diabetes and Alzheimer’s disease. *Biochim. Biophys. Acta* 1792, 482–496.

Zhou BP, Liao Y, Xia W, Spohn B, Lee MH & Hung MC (2001) Cytoplasmic localization of p21Cip1/WAF1 by Akt-induced phosphorylation in HER-2/neu-overexpressing cells. *Nat. Cell Biol.* 3, 245–252.
